# A coarse-grained approach to model the dynamics of the actomyosin cortex

**DOI:** 10.1186/s12915-022-01279-2

**Published:** 2022-04-22

**Authors:** Miguel Hernández-del-Valle, Andrea Valencia-Expósito, Antonio López-Izquierdo, Pau Casanova-Ferrer, Pedro Tarazona, Maria D. Martín-Bermudo, David G. Míguez

**Affiliations:** 1 Centro de Biología Molecular Severo Ochoa, Universidad Autónoma de Madrid, Madrid, 28049 Spain; 2 IFIMAC, Fac. de Ciencias, Universidad Autónoma de Madrid, Madrid, 28049 Spain; 3 Instituto Nicolás Cabrera, Fac. de Ciencias, Universidad Autónoma de Madrid, Madrid, 28049 Spain; 4 Fisica de la Materia Condensada, Fac. de Ciencias, Universidad Autónoma de Madrid, Madrid, 28049 Spain; 5 Centro Andaluz de Biología del Desarrollo, Universidad Pablo de Olavide/CSIC/JA, Carretera de Utrera km 1, Seville, 41013 Spain; 6 Fisica Teórica de la Materia Condensada, Fac. de Ciencias, Universidad Autónoma de Madrid, Madrid, 28049 Spain

**Keywords:** Actomyosin, Coarse-grained, Oscillations, Cytoskeleton, Mathematical models

## Abstract

**Background:**

The dynamics of the actomyosin machinery is at the core of many important biological processes. Several relevant cellular responses such as the rhythmic compression of the cell cortex are governed, at a mesoscopic level, by the nonlinear interaction between actin monomers, actin crosslinkers, and myosin motors. Coarse-grained models are an optimal tool to study actomyosin systems, since they can include processes that occur at long time and space scales, while maintaining the most relevant features of the molecular interactions.

**Results:**

Here, we present a coarse-grained model of a two-dimensional actomyosin cortex, adjacent to a three-dimensional cytoplasm. Our simplified model incorporates only well-characterized interactions between actin monomers, actin crosslinkers and myosin, and it is able to reproduce many of the most important aspects of actin filament and actomyosin network formation, such as dynamics of polymerization and depolymerization, treadmilling, network formation, and the autonomous oscillatory dynamics of actomyosin.

**Conclusions:**

We believe that the present model can be used to study the in vivo response of actomyosin networks to changes in key parameters of the system, such as alterations in the attachment of actin filaments to the cell cortex.

**Supplementary Information:**

The online version contains supplementary material available at (10.1186/s12915-022-01279-2).

## Background

The interactions that govern the dynamics of biological systems take place at size and time scales that can differ several orders of magnitude. At the macroscopic level, computational characterization and analysis of biological processes rely on stochastic or deterministic differential equations. At the atomic resolution level, molecular dynamics (MD) numerical simulations are the main tool but are restricted to very few molecules and short-time scales. In between both scales, models that involve many molecules and/or larger length (typically larger than 10 nm) and time scales (longer than 100 ns) are often based on coarse-grained (CG) approximations [1].

CG models can be defined as mathematical representations of systems made of simplified versions of their more relevant sub-components and interactions [2]. These type of models constitute optimal tools to study system properties that arise from the molecular aspects of the interacting components [3], such as aggregation, polymerization or self-assembly [4]. In the context of the cell cytoskeleton, [5] microtubule formation [6], microtubule mechanics [7, 8], dynamics [9], and interaction with kinesin motors [10] have been addressed computationally using a CG approximation.

The other main component of the cytoskeleton, the actomyosin machinery, has been extensively studied using CG models, such as the assembly [11] and allostery [12] of actin filaments (F-actin) from globular actin (G-actin), its reorganization in networks and bundles in the cell cortex [13], the fluidization of the actin cytoskeleton [14], and other responses under stretching [15]. In addition, Vavylonis et al. propose a numerical model for actomyosin contraction where some of the features of actomyosin network formation are modeled using a Monte Carlo approximation [16].

The effects of crosslinker proteins [17, 18], myosin V, and myosin II [19] have also been studied using a CG approximation [20]. More recently, the CG approximation has been successfully used to model many biological processes [21–30].

One of the most interesting properties of the actomyosin machinery is their ability to undergo periodic oscillations in their concentration [31–34]. These oscillations take place as pulsating reorganization of quasi-two-dimensional networks of actin and myosin in the cell cortex, a specialized layer attached to the inner plasma membrane that plays a central role in cell motility and cell shape control.

This type of autonomous oscillatory dynamics is ubiquitous in biology at many levels, from gene expression [35, 36] to circadian clocks [37] and nervous impulses [38]. At the cellular level, spontaneous mechanical oscillations have been reported in a variety of cell types, including cardiomyocytes [39, 40], fibrils of ordinary skeletal muscles [41–43], insect muscle cells [44], and eukaryote tissue culture cells [45]. In the context of animal development, autonomous oscillations have been shown to govern the dynamics of the segmentation clock during somitogenesis [46, 47], the neurogenesis in the developing cortex [48], or the overall size of the embryo [49]. In addition, the spindle oscillations during asymmetric division in *Caenorhabditis elegans* [50] arise from periodic mechanical coordination of force-generating motors [51]. Apical constriction [52] in ventral furrow cells pulsates during *Drosophila* mesoderm invagination [52]. Periodic contraction and expansion of amnioserosa cells drive tissue movement during dorsal closure [31, 32]. In addition, elongation of the *Drosophila* egg chamber requires periodic oscillations of the actomyosin network present on the basal side of the follicle cells [33, 34].

The basic machinery for these mechanical oscillations relies on the interactions that govern the dynamics of the actomyosin cell cortex. It has been shown that cooperative myosin motors acting over an actin filament oscillate with a frequency of few tenths of Hertzs and around 10 nm of amplitude [53, 54], but the mechanism underlying the much slower and larger actomyosin oscillations responsible for cell shape oscillations is still poorly understood [31, 32]. Recently, several theoretical studies have proposed a combination of mechanical and biochemical interactions for the emergence of periodic constriction of the apical cell cortex of amnioserosa cells during dorsal closure in *Drosophila*. An initial model proposes the interplay between myosin and an unknown signaling molecule that oscillates due to the interaction with myosin combined with coupling between neighboring cells [31]. Recently, several contributions proposed a mechanochemical cell autonomous mechanism based on previous models [45] of pulsating dynamics acting in dividing cells, where coupling between actin turnover and cell area is assumed [55]. Other recent approaches reproduce actomyosin pulsations, by combining the effect of nonlinear actin and myosin turnover, an elastic restoring force and a viscous damper [56]. Other mechanochemical approaches specific to the oscillations in the basal cell cortex in *Drosophila* follicle cells in the developing egg chamber assume cells as springs acting against a potential internal pressure of the egg chamber [57].

We have recently proposed an alternative model for actomyosin oscillations based on aggregation and dissociation of actin filaments mediated by the mechanical action of myosin. The model reproduces the spontaneous oscillations in actomyosin concentration based simply on F-actin aggregation and myosin-induced disassembly of the network [34]. In addition, this simplified model reproduces the experimental features of several mutant conditions in the context of follicle cells in *Drosophila*, such as changes in myosin activity [34] and in integrin levels [58].

In this paper, we use a numerical Monte Carlo approximation to develop a CG model of the actomyosin cortex, based on three main interactions: the polymerization and depolymerization of F-actin from G-actin, the assembly of networks of F-actin attached to the inner cellular membrane mediated by actin binding proteins, and the disassembly of F-actin networks mediated by myosin. Our model successfully reproduces key aspects of the actomyosin cortex, such as polymerization dynamics, filament size distribution, treadmilling, cooperative F-actin assembly, and network formation above a threshold concentration. The model also reproduces the periodic autonomous assembly of the actomyosin cortex observed in many biological systems, as well as the effect in the oscillations due to the changes in the levels of key proteins, such as myosin and integrins.

Despite the highly complex biological regulation of the actomyosin machinery, the model shows that many of the most relevant aspects of the actomyosin cortex can be explained based on simplified coarse-grained interactions between actin, myosin, and crosslinker molecules.

## Results

### The model reproduces the dynamics of F-actin polymerization

In this section, we use the first version of the framework with G-actin as the only ingredient to study the dynamics of F-actin formation, its dependence on key model parameters, and how it compares with well-established experimental observations. Details of how the model is implemented are explained in the “Methods” section.

**Fig. 1.**
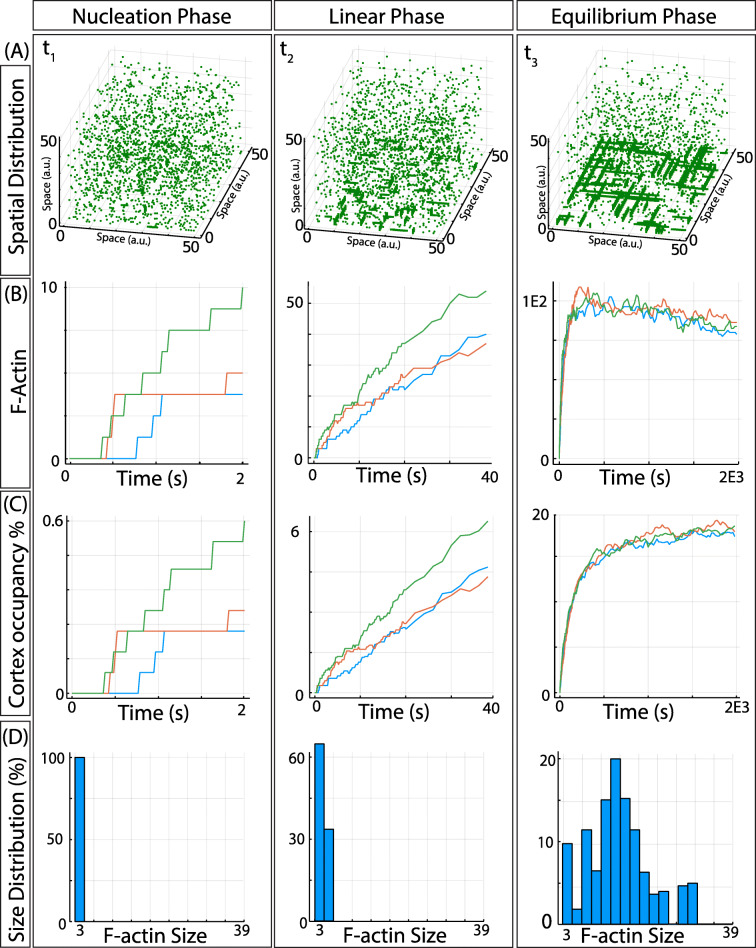
Dynamics of F-actin polymerization. **A** Snapshots of the system at three different simulation times, corresponding to the three regimes: nucleation, polymerization, and equilibrium/reorganization. **B** Number of filaments forming as a function of time during the three regimes. The three lines correspond to three independent runs of the model. **C** Percentage of the nodes in the network occupied by actin molecules as a function of time during the three phases. The three lines correspond to three independent runs of the model. **D** Probability size distribution of F-actin for the three different phases, measured in terms of monomer units. Histograms correspond to three time points of the same numerical realization of the model

The results from numerical simulations of the model (plotted in Fig. 1 for three different time points, time-lapse movie presented as Additional file 1: Movie S1) show that F-actin formation occurs in three distinct phases. The first column corresponds to a *nucleation phase*, characterized by fast assembly and disassembly of short-lived F-actin, composed of very few monomers. In this phase, most of G-actin is still at the cytoplasm (Fig. 1A), while the number of F-actin (Fig. 1B) and the cortex occupancy (Fig. 1C) show an initial lag phase followed by cooperative polymerization.

The size distribution of F-actin polymers has been shown to strongly affect the fluidity and viscosity of polymer solutions, as well as in the cytoplasm, which in turn influences multiple important cellular properties and functions [59–61]. The size distribution of the filaments (measured in terms of monomer units, Fig. 1D) in this phase shows the nucleation of G-actin-free monomers into small filaments. This distribution and dynamics (lag phase and cooperative assembly) reproduces the experimental observations in vitro [62–64].

The second column corresponds to the *polymerization phase*, characterized by F-actin elongation (Fig. 1A), where the amount of F-actin (Fig. 1B) and total G-actin in the filaments (Fig. 1C) increases at an almost linear pace. The size distribution is still monotonically decreasing, with F-actin gradually growing in size, again in good agreement with experimental observations [62–64].

The final *equilibrium/redistribution phase* is characterized by the presence of large filaments and a few short-lived smaller filaments (Fig. 1A). The number of total molecules in filaments fluctuates around a constant value (Fig. 1C), while the number of filaments is slowly decreasing due to the gradual disassemble of these shorter polymers, to the expenses of the further elongation of the longer more stable filaments (Fig. 1B). The size distribution now shows a maximum at intermediate values, evidencing that most G-actin molecules are now part of the filaments of intermediate size, in agreement with experimental observations [62–64].

Interestingly, and despite the simplicity of our model, these three phases (nucleation, polymerization, and equilibrium/redistribution) reproduce the well-known dynamics and the size distribution of the three regimes observed experimentally (Fig. 2 in ref. [65]).

### The model predicts a critical concentration of G-actin for polymerization

**Fig. 2.**
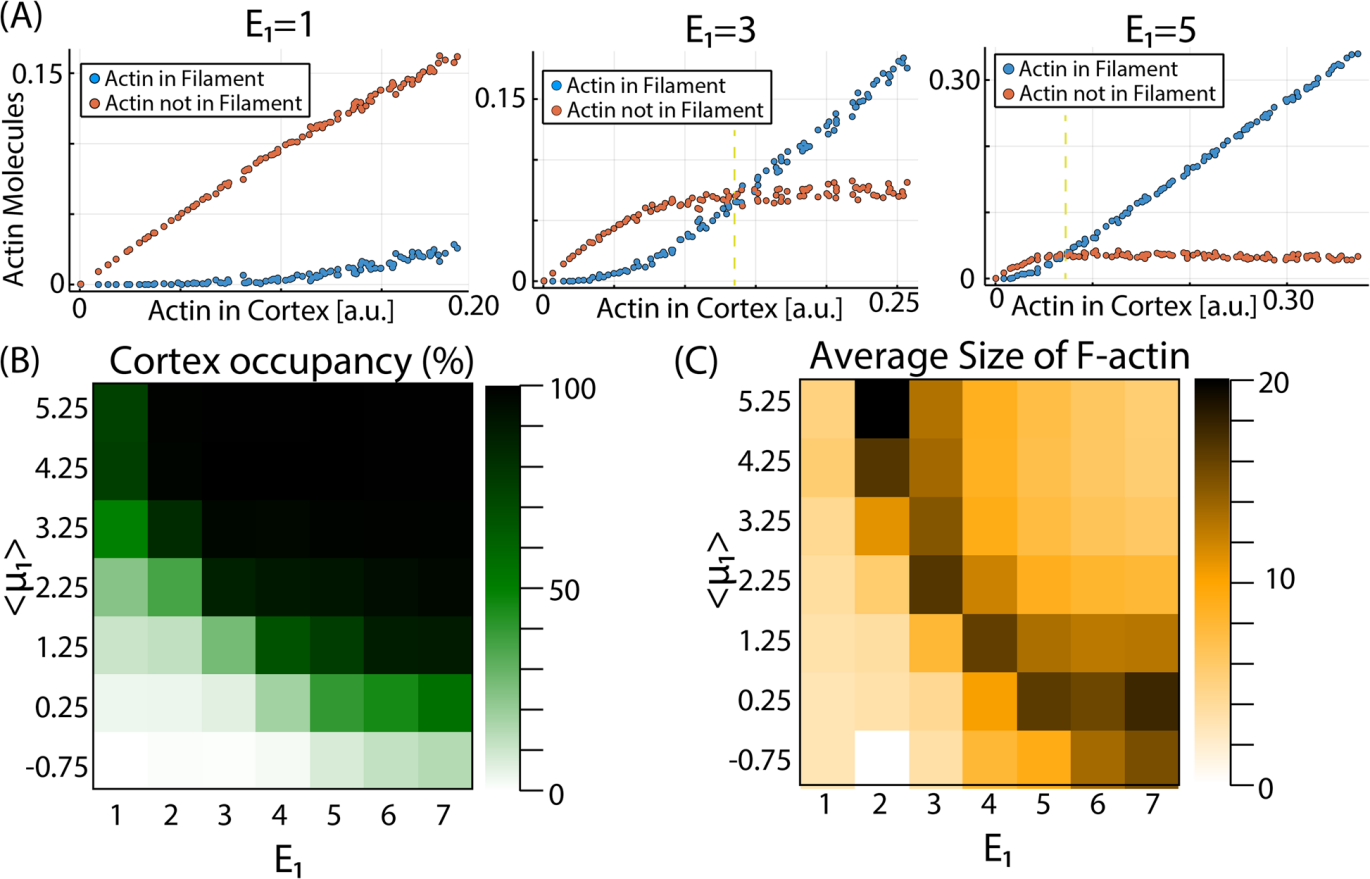
Dependence of F-actin features on model parameters. **A** Dependence of the amount of G-actin in free monomer and polymer configuration on the total amount of G-actin in the system *N*_1,0_ for three different values of *E*_1_. **B** Phase diagram plotting the cortex occupancy for different values of the average < *μ*_1_> and *E*_1_. **C** Phase diagram plotting the average length of F-actin for different values of the average < *μ*_1_> and *E*_1_

Another important observation from experimental work in vitro is shown in the early studies of Oosawa and Kasai [62], where F-actin polymerization occurs only above a critical concentration in G-actin in the system, then increases linearly with the amount of available G-actin. After this transition point, the amount of free monomers should remain constant and independent on the total levels of G-actin in the system (all new G-actin entering the cortex does so as part of F-actin polymers). To test this in our model, we perform independent numerical simulations of the model with different values of the total number of monomers (*N*_1,0_) and monitored the number and features of the F-actin formed at the cortex. The results are shown in Fig. 2A. The model predicts a critical value (yellow dashed vertical line) above which the amount of total G-actin in the cortex (blue dots) increases linearly, while the amount of G-actin not in filament configuration (red dots) remains constant. This critical concentration is more evident for higher values of the link energy of G-actin molecules to the inner plasma membrane *E*_1_ (right panel in Fig. 2A).

The presence of a critical concentration of G-actin can be interpreted in terms of a phase separation phenomenon, similar to gas-liquid condensation (free monomers correspond to gas molecules, and polymers correspond to liquid). This way, F-actin starts to form (liquid phase) when the number of free monomers (gas molecules) reaches a critical density, and further increasing of the total number of molecules results in increases in the liquid phase under a constant density of gas [62].

### The model allows us to study the dynamics of F-actin for different experimental conditions

It is well known that some key properties of the inner membrane cortex depend on the parameters such as concentration of G-actin and affinity towards the membrane, but these parameters are difficult to modulate in vivo. In this section, we take advantage of our framework to study these dependencies directly by changing the parameters of the model and monitoring how the polymerization and depolymerization of F-actin are being affected. The results are shown in Fig. 2B, C.

At the equilibrium, the model shows robust polymerization for a wide range of parameter values, with the total occupancy of the cortex (Fig. 2B) increasing as we increase the average value of the potential < *μ*_1_> (that regulates polymerization) and the energy of the link *E*_1_ (that regulates depolymerization).

A more interesting result appears when the average size of F-actin is computed (Fig. 2C). We see that, for intermediate values of *E*_1_ and < *μ*_1_>, the formation of F-actin is maximized, and large polymers dominate versus smaller polymers and free monomers *N*_1,0_, in agreement with previous studies [63]. When we compute the amount of G-actin molecules distributed in F-actin of different sizes, we observe an exponential distribution (Additional file 2: Fig. S1), as predicted in earlier studies [66].

In conclusion, despite the simplicity of our coarse-grained approach, the model is able to capture many key aspects of F-actin polymerization: its dynamics, the existence of a critical concentration of G-actin, the size distribution in steady state, and how it depends on free monomer concentration. The phase diagrams also show that increasing < *μ*_1_> is equivalent to increase < *E*_1_> (phase diagrams are symmetric respect to the diagonal). This is true when monitoring the concentration of F-actin (Fig. 2B) and its average size (Fig. 2C), but not for other features of F-actin, as we will see later. Also, we can also observe that values around *E*_1_=5, < *μ*_1_> =−1.25 result in the most efficient polymerization (i.e., many large filaments at low cortex occupancy).

### The model reproduces filament treadmilling

In conditions of constant concentration of G-actin, F-actin grows principally at the barbed end and shortens at the pointed end. When the rate of polymerization and depolymerization is balanced in a given F-actin, it produces an apparent displacement of F-actin known as treadmilling, which is essential for cell motility and other cellular processes [67, 68].

**Fig. 3.**
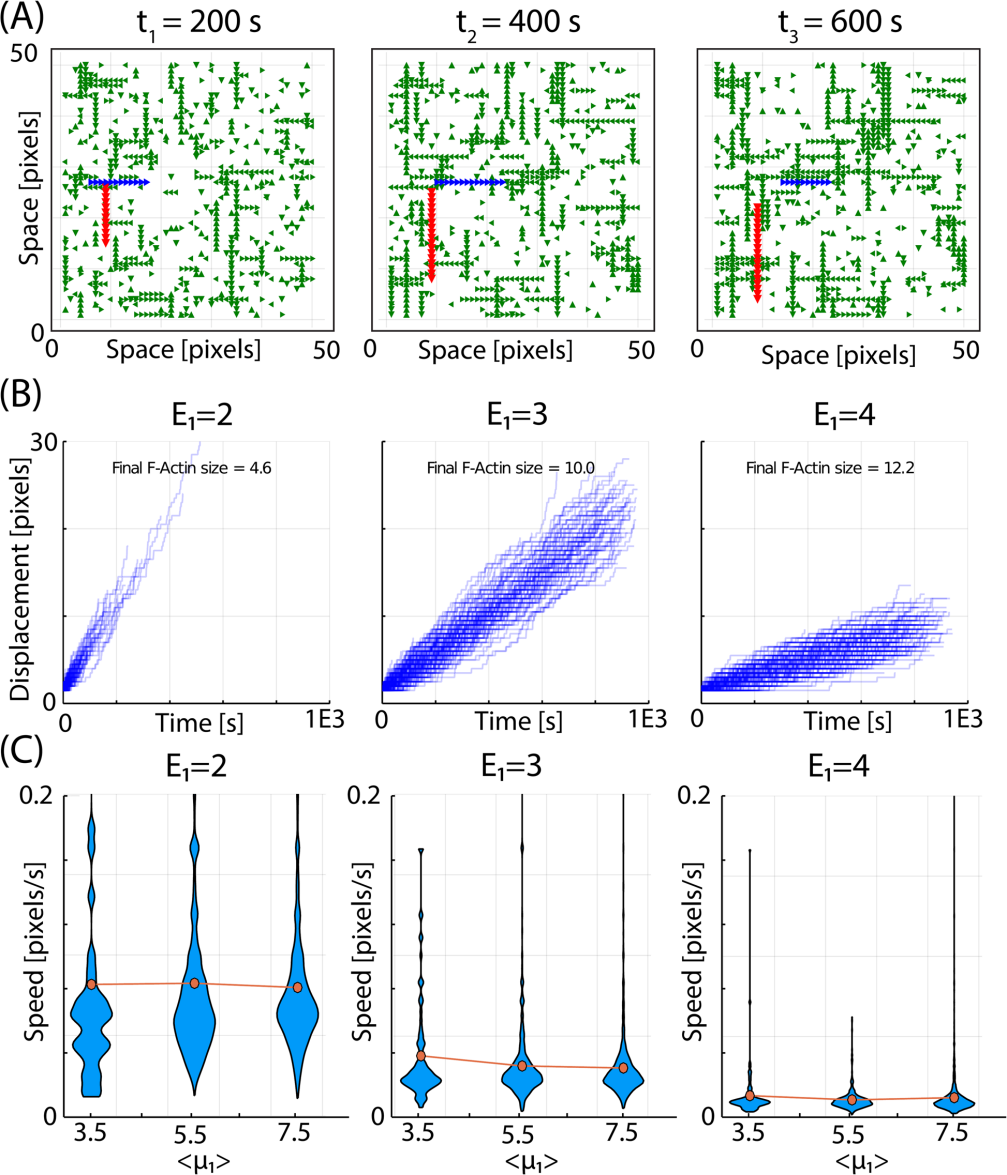
The model reproduces F-actin treadmilling. **A** Snapshots of the cell cortex showing two filaments (highlighted in red and blue) moving in different directions due to directional treadmilling. **B** Displacement of all filaments in a simulation showing the dispersion in velocity values for different values of energy *E*_1_. **C** Dependence on the velocity of filaments on the potential < *μ*_1_> for different values of the *E*_1_

To study the process of treadmilling in our model, we perform time-lapse simulations where filaments can be tracked to monitor their position. Snapshots of the cortex at three time points of a given simulation are shown Fig. 3A, where we highlighted two filaments that move up to down (red) and right to left (blue). Filament treadmilling can be seen also in Additional file 1: Movie S1.

Treadmilling in our model occurs because polymerization is probabilistic, while depolymerization is time-dependent (i.e., the effective probability of disassembly increases with time). This way, at some average F-actin length, the rate of polymerization balances the rate of depolymerization, resulting in the apparent directional displacement of the filaments.

Figure 3B plots the displacement overtime of the filaments for three simulations with different values of *E*_1_, showing that the average speed of the filaments depends strongly on this parameter.

Figure 3C plots the speed of the F-actin for the three energies and for different values of < *μ*_1_>. Interestingly, the speed of treadmilling does not depend strongly on < *μ*_1_> (which strongly affects the size of F-actin, as shown in the previous section). This means that changes in *E*_1_ modulate both F-actin speed and length, while changes in < *μ*_1_> only affect the average length while maintaining the average displacement velocity.

Figure 3B, C illustrates the strong variability for the speed of the different F-actin in the same simulation. An analysis of the speed of the filaments and their length (Additional file 3: Fig. S2) shows that the shortest filaments move faster than the average, while the speed of larger filaments does not depend on their length.

### The model reproduces cooperative network assembly

**Fig. 4.**
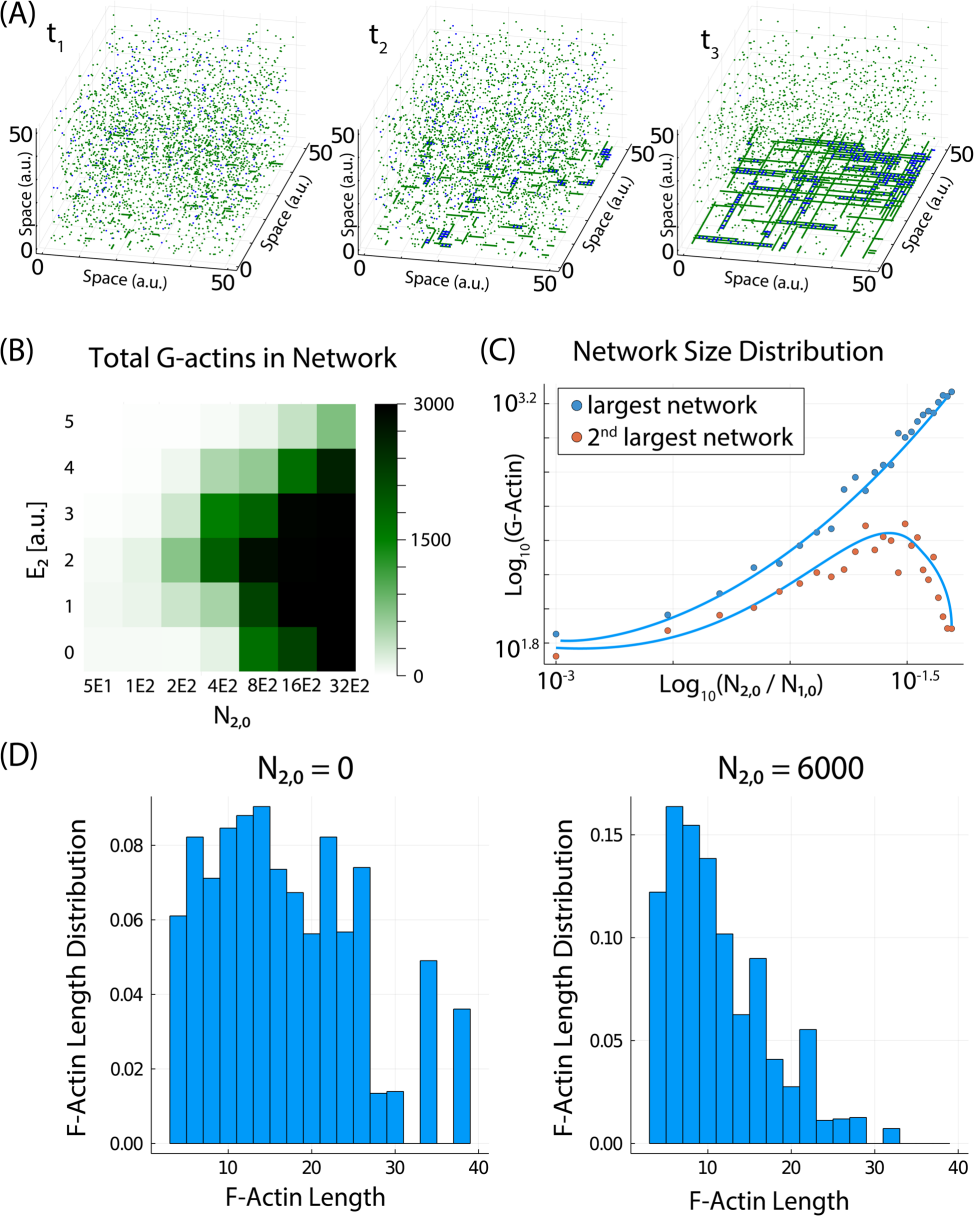
The model reproduces the dynamics of network formation. **A** Snapshots of system for different time points of a given simulation. ACs are added at *t*_1_. **B** Phase diagram showing the average network size at equilibrium for different values of *E*_2_ and *N*_2,0_. **C** Size of the largest and the second largest networks for different ratios of *N*_2,0_/*N*_1,0_. **D** Length distribution of filaments without (left) and with (right) ACs

In this section, we modify our framework to study the effect of actin crosslinkers (ACs) in the configuration of the inner plasma membrane. Details of how ACs are modeled are explained in the “Methods” section. Snapshots of the system in the presence of ACs are shown in Fig. 4A for three different time points (time-lapse movie shown as Additional file 4: Movie S2), showing an initial state with several small networks that eventually grow in size by fusing to other networks and by increasing the length of the F-actin.

It has been shown experimentally that the formation of F-actin networks and bundles [69] only takes place above a critical concentration of ACs [70–72]. To study this, we computed a phase diagram (Fig. 4B) where the average amount of monomers in the cortex is computed for different values of the link energy between ACs and actin molecules (*E*_2_) and the total amount of ACs (*N*_2,0_). The plot shows that, for a given value of the energy *E*_2_, the formation of networks only occurs above a critical value of the concentration of ACs, in agreement with experimental observations [70–72].

Interestingly, for a given concentration of ACs, the size of the network is maximized at intermediate values of the energy *E*_2_. This is due to the fact that low-energy links are very unstable and are broken rapidly (F-actin is unstable and does not has time to grow in size). On the other hand, very strong links are almost never removed, so reorganization of the network cannot take place (small filaments are so stable that block the growth of other filaments). Therefore, intermediate values of *E*_2_ maximize network size.

Previous studies also focus on the competition between networks for different amounts of ACs [73], showing that the largest network increases at the expenses of the decrease of the second largest network when the amount of ACs is increased. This competition also takes place in our model (Fig. 4C), in agreement with the previous studies [73].

Network formation is a highly cooperative mechanism [13] (as the network grows, the probability of successful adding more ACs also increases). The dynamics of incorporating the molecules into the networks (Additional file 5: Fig. S3) shows an initial very fast incorporation of ACs and G-actin as soon as the ACs are added into the system.

Another important experimental observation measures the reduction in the width of the length distribution of F-actin after addition of ACs [74]. Our model also shows the same reduction of the standard deviation in the distribution, in the presence of ACs (Fig. 4D).

In conclusion, our model reproduces many key experimental features of F-actin networks, such as the cooperative assembly, the threshold concentration of ACs, the competition between networks, and the homogenization of the length of the filaments in the presence of ACs.

### The model reproduces actomyosin oscillations

**Fig. 5.**
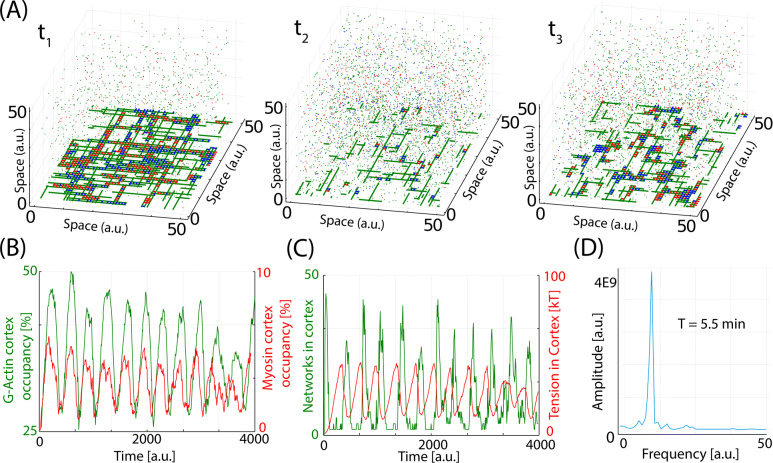
The model reproduces actomyosin oscillations. **A** Snapshots of the system for three different time points of a given simulation (green is G-actin, blue is ACs, red is myosin). **B** Temporal evolution of total actin and myosin in the cortex. **C** Temporal evolution of tension (red) and number of bundles (green)

In this section, we use our GC framework to study the dynamics of actomyosin when we include tension-induced disassembly of the F-actin, mediated by the mechanical action of myosin. Details of how myosin and F-actin load is introduced in the model are explained in the “Methods” section (in brief, myosin attaches to F-actin with a link energy of *E*_3_ and produces a mechanical force as long as it remains attached to two F-actin). Snapshots of the system at three time points of the same simulation are shown in Fig. 5A (time-lapse movie shown in Additional file 6: Movie S3). In the first time point (left panel), a large percentage of the molecules are at the cortex, as part of a network. Then (central panel), the network is disassembled and the molecules are mainly at the cytoplasm. The next time point (right panel) shows the actomyosin cortex starting to form again.

The total amount of G-actin (green) and myosin (red) in the cortex during these oscillations is plotted in Fig. 5B, where we can see periodic changes in both levels in the form of autonomous oscillations with a defined period. Figure 5C plots the total tension in the cortex (red line) and the number of simultaneous networks (green line). Interestingly, the tension increases in anti-phase with number of networks (i.e., as the largest network takes over the system, the tension increases until some of the filaments reached their threshold in tension). The release of tension is also much faster than the disassembly of the network, and the difference between these two time scales is at the core of this oscillatory dynamics. Figure 5D plots the Fourier transform of the temporal evolution in the number of G-actin at the cortex, with a clear peak evidencing the periodic behavior. The period of oscillation is around 5.5 min, which is similar to the values measured in vivo [31, 45, 55–57].

Previous experimental studies [34] show that actomyosin oscillations accelerate, decrease in amplitude, and become less periodic and more stochastic when the amount of active myosin is increased. To test this situation, we compared in Additional file 7: Fig. S4 the oscillations for low (*M*_3,0_=200, panel A) and high (*M*_3,0_=4000, panel B) levels of myosin. We can see that, as we increase the myosin concentration, oscillations are smaller, faster, and more stochastic, in agreement with the experimental observations [34].

### The attachment of actin molecules to the cortex modulates the network architecture and dynamics

**Fig. 6.**
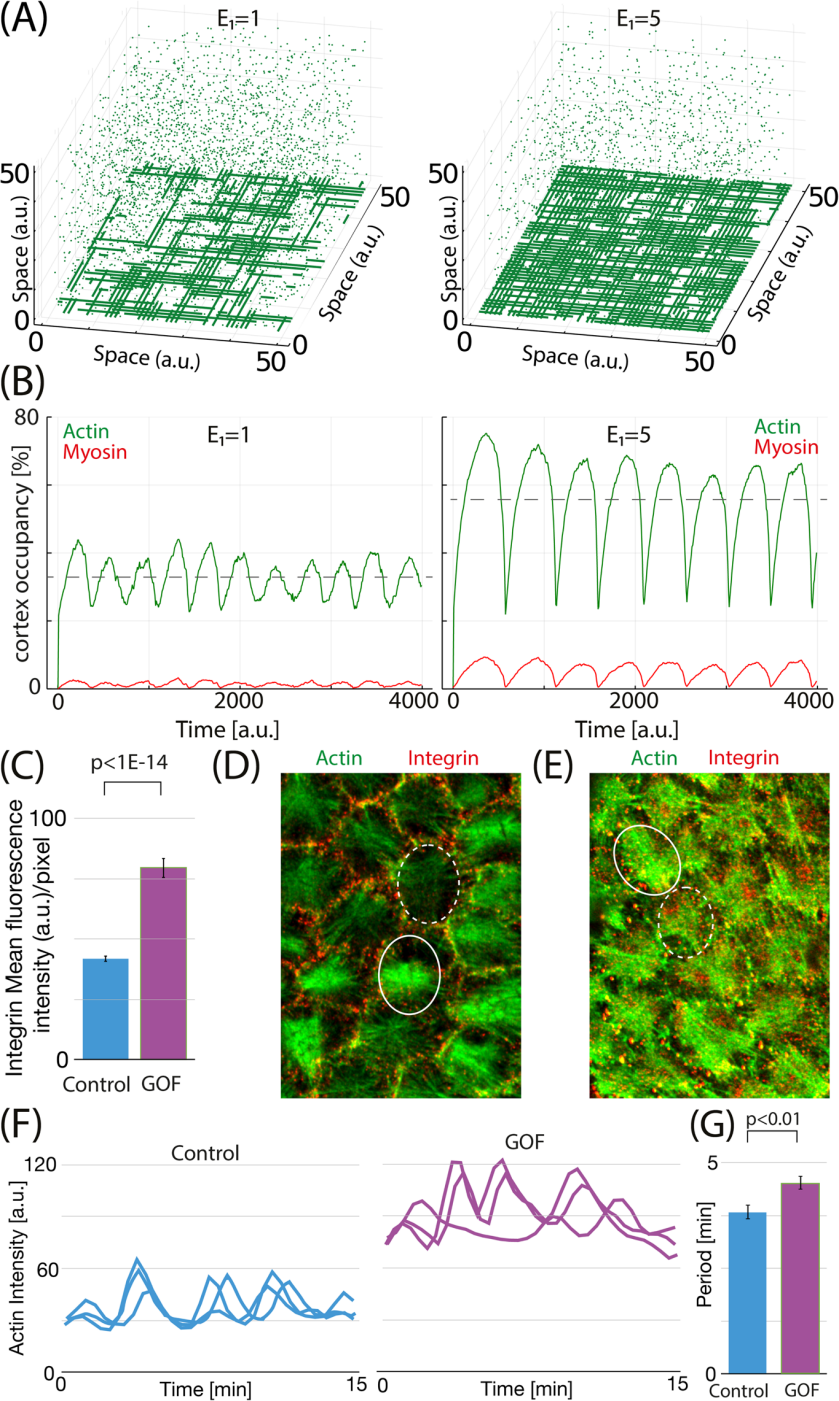
The model predicts the effect of integrin overexpression. **A** Snapshots of the system for two different values of *E*_1_ (green is G-actin, snapshot taken at the point of maximal value of occupancy in a oscillation). **B** Temporal evolution of total actin and myosin in the cortex for the two values of *E*_1_ tested. Dashed gray lines show the average levels of actin. **C** Mean fluorescent intensity of integrins in stress fibers of follicle cells in control and *α*PS1; *β*PS overexpression (labeled as gain of function (GOF)) conditions (*N* = 11 for each condition). **D**, **E** Confocal images of the basal surface of follicle cells for control (**D**) and GOF (**E**) conditions expressing the F-actin live marker Ubi-LifeActinGFP stained for anti-GFP (green) and anti-BPS integrin (red). Circles denote follicle cells with the highest (solid line) and the lowest (dashed line) levels of F-actin fluorescence. **F** Quantification of the dynamic changes of basal F-actin in three individual follicle cells for control (left) and GOF (right) conditions. **G** Basal F-actin average oscillation period for control and GOF conditions (*N* = 11 for each condition)

In previous sections, we have corroborated that the model reproduces many already established important aspect of the actomyosin machinery. In the present section, we proceed to test the model and its ability to reproduce the effect of modulating the attachment of F-actin to the cortex. This effect is studied by varying the attachment strength of the actin molecules to the cortex (*E*_1_) and are summarized in Fig. 6A, B. Our results show a high dependence of the F-actin architecture and network dynamics on the attachment strength of the actin molecules to the cortex and that increasing *E*_1_ results in a more dense cortex with F-actin of larger length (Fig. 6A, snapshots taken at the maximum of the oscillation). Focusing on the oscillatory dynamics, both amplitude and period of the oscillations increase when *E*_1_ is increased (Fig. 6B).

To modulate experimentally the attachment of F-actin to the inner cell membrane, we take advantage of integrins, trans-membrane proteins that mediate cell-to-matrix and cell-to-cell adhesion, with important roles in cell adhesion, migration, proliferation, and survival [75]. Integrins are known to be coupled to actin molecules in the cortex through physical linkage [76] through actin-binding molecules, such as talin [76], and they have been shown to shape the architecture and mechanical properties of actomyosin networks [58]. Using the actomyosin fibers present on the basal side of *Drosophila* follicle cells (FCs) as a well-established model to study actomyosin dynamics [33], we and others have previously shown that integrins are required for the correct recruitment, formation, and maintenance of these basal F-actin fibers [58, 77].

Integrins are heterodimeric proteins composed of an *α* and a *β* subunit, and both are required to transport integrins to the surface. Two integrins are expressed in FCs: the *α*PS1 *β*PS (PS1) and the *α*PS2 *β*PS (PS2). Here, to study the effect of integrin levels, we use the UAS/Gal4 system [78] to overexpress both *α*PS1 and *β*PS subunits of the *PS*1 hetero-dimer simultaneously, using the traffic jam Gal4 line (tj-Gal4, [79]) driver (tj>PS1).

The comparison of the dynamics and configuration of the actomyosin cortex in conditions of control and *α*PS1; *β*PS overexpression (labeled as gain of function (GOF)) is summarized in Fig. 6C–G. As a first control, Fig. 6C shows that in GOF conditions, there is an statistically significant increase in the amount of integrin attached to the actomyosin filaments in the cortex.

In addition, the amount of F-actin accumulated in fibers in GOF conditions was also higher than in control follicle cells (Fig. 6E, G). These results suggest that increasing the amount of integrins in FCs is an efficient way to increase the amount of F-actin fibers at the cortex, similar to what happens in the simulations when increasing the value of *E*_1_ (Fig. 6A). Interestingly, we found that the average length of F-actin filaments of FCs overexpressing PS1 increases, compared to control conditions, in full agreement with the model predictions (Fig. 6A, E).

Next, we focus on the dynamics by quantifying the effect of elevated integrin levels in the oscillatory behavior of F-actin. To do that, we performed live time-lapse imaging of egg chambers for both control and GOF conditions expressing the in vivo marker for F-actin, Ubi-LifeAct-GFP (see the “Methods” and [58]). We found that the basal F-actin of tj>PS1 FCs underwent periodic fluctuations (*n* = 8, Fig. 6G, Additional file 8: Movie S4 and Additional file 9: Movie S5). However, in contrast to the marked and periodic changes observed in control FCs, the oscillation period in tj>PS1 FCs showed a high degree of variability, and both maximum and minimum (solid and dashed circles, respectively) show higher intensity levels in GOF conditions, compared to control (Fig. 6D, E). This suggests that, in GOF conditions, the actomyosin complex is not being fully disassembled during the course of the oscillation, compared to a more complete disassemble in control FCs. Quantification of the pulsation frequency revealed that the period of tj>PS1 FCs was 17% higher than that of controls, in agreement with model predictions.

On the other hand, the experiments do not show an increase in oscillation amplitude, while the model does show larger differences between maximum and minimum levels of actin. We hypothesize that these differences can be caused by the reduced temporal resolution of the experimental data compared to the model. To test this, we produced similar plots of Fig. 6B but using a moving average filter to reduce resolution. The results shown in Additional file 10: Fig. S7 show that, if resolution is lowered, the lower part of the oscillation is filtered out, and the increase in *E*_1_ results basically in a net increase in the average levels of the actin in the cortex, more similar to the effect observed experimentally (Fig. 6F).

In conclusion, our model does not only reproduces well-known features of the actomyosin cortex, but can also reproduce some important features of basal F-actin oscillations in wild type and in experimental perturbations.

## Discussion

In this paper, we presented a GC model of the actomyosin cortex that includes interactions between molecules and the inner plasma membrane. The most complex task in the design of a useful GC model is to select the main relevant ingredients that govern the dynamics of the system. The full version of the model is based on only three basic aspect of the actomyosin machinery: (1) polymerization and depolymerization of F-actin from G-actin at the inner cellular membrane, (2) supramolecular organization of F-actin in networks mediated by ACs, and (3) reorganization of the network mediated by myosin. Despite this highly simplistic approach, our model successfully reproduces many of the most relevant processes involving F-actin: (1) F-actin formation occurs in three regimes (nucleation, linear, and equilibrium/redistribution), (2) size distribution in these three regimes, (3) threshold for F-actin polymerization, (4) treadmilling, (5) competition between networks, (6) cooperativity and periodic assembly and disassembly of actomyosin networks, and (7) the effect if integrin in the dynamics and configuration of the actomyosin cortex.

In the present model, autonomous oscillations arise from the interplay between these cooperative assembly of the actin cortex coupled to myosin-induced disassembly, which is also the process that drives the oscillations in our previous model [34]. In both systems, oscillations become less periodic and more stochastic when the amount of active myosin is increased. This occurs because the network collapses before being fully assembled; therefore, there is no global oscillation and the network forms and disassembles more locally, and the net result at the system level is a less periodic behavior.

The model assumes several important simplifications. It assumes that the inner plasma membrane is a two-dimensional grid where F-actin molecules of the same orientation are not allowed to overlap (cortex thickness has been estimated to be around a few hundred nm [80], while diameter of F-actin is around 10 nm). In addition, F-actin is often composed by many more G-actin subunits than in our model. Also, we have not incorporated the fact that F-actin can grow from both barbed and pointed ends, but the dynamics of growth in the barbed end is much faster than that in the barbed end. This can be easily implemented as an extension of our model, although we expect that the conclusions derived from the model will be equivalent.

The model also assumes that molecules in the reservoir diffuse instantaneously compared to two-dimensional diffusion while in the cortex. In addition, G-actin does not diffuse while in the cortex, and it is assume to be linked to the membrane. Another simplification is to assume ATP in excess in the reservoir, so the effect of ATP is not explicitly included in the model for simplicity. In the same direction, we do not model explicitly the interaction between myosin and ATP. Also, the grid only has two directions, which is a strong simplification compared to the experimental situation. A potential extension of the model is to allow another direction by setting up the grid as an hexagonal lattice.

Based on their effect in cortex occupancy (Fig. 2B) and polymer size (Fig. 2C), it seems that potential and link energy are equivalent (i.e., increasing one is equivalent to increasing the other). Interestingly, they are not at all equivalent in terms of the dynamics of the filaments (Fig. 3C). Since the potential affects the entrance into the grid and polymerization, and *E*_1_ controls the depolymerization and the release, we can see that increasing < *μ*_1_ > has almost no effect in the threadmilling speed, while increasing *E*_1_ strongly slows down the displacement (G-actin monomers are linked stronger to the grid, so they are more difficult to remove). This means that, playing with both parameters, the model can produce filaments of a given length (by dialing the potential <*μ*_1_>) and a desired speed (by dialing the energy *E*_1_).

Treadmiling in our models is nondirectional, contrarily to the directional dynamics observed in filopodia and lamelopodia [68]. This is due to the lack of regulatory proteins in our model, such as ADP/cofilin and caping subunits that break the symmetry and favor directional growth of the filaments.

Several studies show that the architecture of the network ultimately determines the dynamics of the interaction of actin with myosin [81]. This architecture depends on the different types of ACs that are known to link F-actin. Implementing other crosslinkers with other binding characteristics will result in different scenarios and will be part of a follow-up study to this one.

Finally, the dynamics of polymerization of nonmuscle myosin is also a complex and multi-step process that it is not fully characterized. For simplicity, our model simply assumes myosin already in its filament form.

All these limitations can be easily addressed in a more detailed version of the model, using high-performance computers, but we believe that the relevance, impact, and usability of a model are higher when the main features can be obtained with conventional hardware. In addition, the model is not designed to reproduce the small details of the actomyosin cortex but to illustrate the sets of minimal interactions that set the most important characteristics at the system level.

When comparing to the experimental data in the last section of the results, the experiments show increased stochasticity in the oscillatory dynamics when the integrin levels are increased. This is not the case in the model, where a single network is formed in every oscillation, and oscillations are very periodic independently on the level of *E*_1_, at least for these parameter values. This highlight one of the clear limitations of the model, since in the real systems, many actomyosin sub-networks are formed simultaneously in these conditions, resulting in a less regular behavior (the same occurred when modulating the myosin levels experimentally [34]). Also, the experiments shown F-actin of length larger than the average cell diameter, so maybe the stochasticity arises from coupling between cells. These qualitative observations, and the potential mechanical coupling between cells, remain to be studied in detail in the future.

## Conclusion

The study of the fundamental properties of transiently cross-linked networks is highly complicated due to the multiple space and time scales involved [82]. Systems such as the actomyosin cortex, where features emerge as system properties due to interactions between many players, present a problem that can be optimally approached using GC models. One limitation of these CG models is that, due to the minimal set of players and interactions, they are not suited to study the details of the biological system in question. On the other hand, they are very useful to isolate the most important players and interactions that are at the core of basic biological phenomena [83].

In our model, myosin motors and crosslinkers interplay with F-actin polymerization and affect the dynamics of the network, its conformation, its dynamics, and its mechanical properties. Our model can be used to study the dynamics of polymerization, the basis of treadmilling, and the cooperative phenomena that drive polymerization and network formation, the thresholds, and the critical concentrations. In addition, our model allows us to study how tension and adhesion shape actomyosin networks in the cell cortex, resulting in periodic oscillations that reproduce many aspects that have been observed experimentally. Our framework shows that, despite the complexity of the actomyosin cortex, many of its key features can be explored and reproduced from very few well-characterized interactions at the molecular level.

## Methods

### Modeling framework and model assumptions

Numerical simulations of the actomyosin cortex are performed using a finite multi-compartment GC framework. The dynamics and the probability distribution of the different micro-states of the systems are represented statistically by a semi-grand canonical ensemble approach. Historically, the most used ensembles in these type of coarse-grained models are canonical (fixed number(s) of molecules) and grand-canonical (fixed chemical potential *μ* also known as macro-canonical, or *μ*VT ensemble). The traditional text-book distinction between canonical and grand-canonical (fixed *μ*) is open to a variety of semi-grand canonical ensembles (ref [84] and references therein), where a link between *μ* and N can be established. In many cases, such an in mixtures and open systems with a finite number of molecules available, the semi-grand canonical ensemble constitutes a more realistic representation [85, 86].

In our system, the inner cellular membrane is defined as a two-dimensional region where the number of molecules is not fixed, but the total number of molecules available is limited. In addition, the system is composed by several types of particles that coexist and interact (in our case, G-actin, actin crosslinkers (ACs), and myosin). Based on these characteristics, the semi-grand canonical approach is the optimal choice [85, 86].

The code is written in Julia language [87], and the following libraries have been used: LightGraphs, FFTW, Plots, Random, Printf, Dates, Statistics, StatsPlots, KernelDensity, and GLM.

The model is solved numerically using a Monte Carlo (MC) computational algorithm that relies on repeated random sampling to obtain numerical results. The dynamics is determined by the Metropolis method [88], used to evaluate if a given transition between an “old” and a “new” state of the ensemble is accepted. The algorithm proceeds as follows: a position [ *x,y*] at the cortex grid is chosen at random, and a change in the state in the system due to an insertion or removal of a molecule in this particular position [ *x,y*] is evaluated. This process is then repeated for each of the three particle types at every time step. Parameters used to produce the figures of the present manuscript are listed in Table 1. In the following paragraphs, we explain the biological motivation behind the design of the framework, and how these features are implemented at the computational level.

**Table 1:** Values for the parameters used in the panels

Parameter values for figures												
Figure	*μ*_1,0_	*N*_1,0_	*γ*_1_	*E*_1_	*μ*_2,0_	*N*_2,0_	*γ*_2_	*E*_2_	*μ*_3,0_	*N*_3,0_	*γ*_3_	*E*_3_
Figure 1	− 5	3000	2	4	–	–	–	–	–	–	–	–
Figure 2A	− 5	Label	2	Label	–	–	–	–	–	–	–	–
Figure 2B, C	Label	Label	2	Label	–	–	–	–	–	–	–	–
Figure 3A	− 4	2000	1	4	–	–	–	–	–	–	–	–
Figure 3B	− 3	5000	1	Label	–	–	–	–	–	–	–	–
Figure 3C	Label	5000	1	Label	–	–	–	–	–	–	–	–
Figure 4A	− 5	3000	2	3	− 5	300	2	3	–	–	–	–
Figure 4B	− 2	3000	2	3	− 2	Label	2	Label	–	–	–	–
Figure 4C	− 2	Label	2	3	− 2	Label	2	3	–	–	–	–
Figure 4D	− 4	6000	2	3	− 4	Label	2	3	–	–	–	–
Figure 5A	− 3	5000	1	3	− 5	500	1	3	-5	250	1	3
Figure 5B–D	− 3	5000	1	1	− 5	3000	1	3	-5	500	1	3
Figure 6A, B	− 3	5000	1	Label	− 5	3000	1	3	-5	500	1	3
Figure S1	− 5	3000	2	4	–	–	–	–	–	–	–	–
Figure S2	− 3	2000	1	3	–	–	–	–	–	–	–	–
Figure S3	− 5	3000	2	3	− 2	300	2	3	–	–	–	–
Figure S4	− 3	2500	1	5	− 5	1250	1	3	− 5	Label	1	3
Figure S5D	Label	2500	1	–	–	–	–	–	–	–	–	–
Figure S5E	0	Label	1	–	–	–	–	–	–	–	–	–
Figure S5F	0	2500	Label	–	–	–	–	–	–	–	–	–

#### Cortex

The actomyosin cortex is a pseudo-two-dimensional mesh of actin filaments, actin crosslinkers, and myosin motors attached to the inner plasma membrane by anchor proteins. The contractile tension imposed by myosin in the network drives important changes in the shape of cells [80]. Our model represents this inner plasma membrane as a two-dimensional grid of fixed size where molecules can interact (see Additional file 11: Fig. S5A for an illustration of the system). Each node of the grid can accommodate one G-actin molecule in vertical orientation (north, south) and one in horizontal orientation (east or west), to allow overlapping of perpendicular filaments. Bonds between molecules are simplified as contact interactions (i.e., molecules can interact if they are in neighboring positions of the grid). The strength of the interactions can be modulated to produce different scenarios. For simplicity, molecules are not allowed to move or rotate when in the grid. G-actin molecules occupy the center of the pixels in the grid, and ACs and myosin occupy the space in between pixels, linking adjacent monomers in neighboring pixels.

#### Cytoplasm

Directly attached the two-dimensional grid, the model assumes a finite three-dimensional reservoir, which mimics the role of the cytoplasm, where molecules diffuse freely (Additional file 11: Fig. S5A). For simplicity, diffusion or movement in the cytoplasm is assumed to be much faster than the process of attaching and detaching to the cortex. Therefore, the cytoplasm is simply modeled as a zero-dimensional reservoir. The total number of molecules of each type in both regions (cortex + cytoplasm) is maintained constant throughout the simulation.

#### Transitions from cytoplasm to cortex

During the simulation, three types of molecules (*i* = 1 for G-actin, *i* = 2 for ACs, *i* = 3 for myosin) move from the cortex to the cytoplasm (Additional file 11: Fig. S5B-C) based on the value of a chemical potential, *μ*_*i*_, computed as a generic function with the following form.

$$\mu_{i} = \mu_{i,0} - \gamma_{i}*\text{log}\frac{N_{i}}{N_{i,0}} $$

where *μ*_*i*,0_ is a reference potential for each molecule type, *N*_*i*,0_ is the total number of molecules of type *i* in the system, and *N*_*i*_ is the number of molecules of type *i* in the grid at a given time point. Parameter *γ*_*i*_ is a form factor that modifies the shape of the potential function.

Therefore, values of *μ*_*i*_ higher than 0 favor the transition of molecules of type *i* from the cytoplasm to the cortex, while values lower than 0 disfavor it. Plots of the potential function for different values of *μ*_*i*,0_,*N*_*i*,0_, and *γ*_*i*_ are illustrated in Additional file 11: Fig. S6D-F.

When the position [ *x,y*] being evaluated is empty, the value $\phantom {\dot {i}\!}P^{+}=e^{\mu _{i}}$ for a molecule of type *i* is computed, and the Metropolis method is used to accept or reject the incorporation of the molecule (a random number *P*_rand_ from a uniform distribution between 0 and 1 is compared with the value of *P*^+^. The change in the state of the system is accepted if *P*_rand_ < *P*^+^).

#### Transitions from the cortex to the cytoplasm

When the position [ *x,y*] being evaluated is occupied by a molecule of type *i*, the value *P*^−^=*e*^−*E*^ is calculated, being *E* the sum of the energy of all the links between this particular protein of type *i* and the other proteins of the cortex. Again, the Metropolis method is used to accepted or rejected the change (i.e., if *P*_rand_ < *P*^−^, the change is accepted).

#### Time calibration

To calibrate the temporal scale of the model, we use in vivo measurements of the polymerization of F-actin in the cortex of mammalian cells (Fig. 1C in [89]), where authors estimated the time to reach equilibrium in the F-actin networks around 18 min. This value is used as a reference to establish a correlation between time iterations and real units of time, to be able to compare with experimental data.

In addition, the speed of the simulations in these type of systems depends by definition also on the size of the grid (due to the random sampling strategy). Therefore, the time is also calibrated by dividing it directly by twice the area of the grid (since each point in the grid can accommodate two G-actin molecules simultaneously, one in vertical and other in horizontal orientation).

#### F-actin polymerization and depolymerization

G-actin is a globular multi-functional and polar protein present in all eukaryotic cells. In the presence of ATP, G-actin can polymerize to form F-actin, a semi-flexible directional micro-filament composed of several thousand actin monomers [90]. F-actin polymerization is mainly directional, i.e., new G-actin monomers are incorporated at one end of the filament (barbed end), while G-actin is released from the other end (pointed end), driven by the ADF/cofilin (AC) family of proteins [66]. In addition, actin molecules are maintained at the cortex by links to anchor proteins at the inner membrane layer, such as the integrin family of cell adhesion molecules [58, 75]. This proteins are linked to F-actin by specific actin-binding proteins, such as talin, vinculin *α*-actinin, and others [76].

Previous studies define the nucleus for F-actin polymerization as the minimum number of aggregated monomers that is favored to grow [62–64]. Based on this definition, the polymerizing nucleus in our model is composed of three monomers (the bond of the last G-actin is destabilized), and therefore, F-actin is defined when formed by three or more G-actin monomers. This is in agreement with recent experimental observations [91].

Polymerization in our model is assumed to occur only at the cortex, as follows: (a) if the sampled position in the grid [ *x,y*] is empty, the successful incorporation of a G-actin from the cytoplasm to this position in the grid is evaluated using $\phantom {\dot {i}\!}P^{+}=e^{\mu _{1}}$ and the metropolis method; (b) if the incorporation is accepted, the orientation of the molecule in the grid is chosen at random between four possible configurations: north, south, east, or west; (c) when a G-actin molecule enters the grid in front of a F-actin with the same orientation, a bond (with energy *E*_0_) is formed, and the free monomer gets incorporated as part of the filament; and (d) attachment of G-actin molecules to the inner plasma membrane [58, 75, 76] is also established with an energy *E*_1_. This way, the total energy of the attachment of a given F-actin to the inner cell membrane can be computed as the sum of all its links (i.e., *E*_threshold_= *E*_1_×*L*, being *L* the length of the filament).

Depolymerization of F-actin takes place as follows: (a) if the position [ *x,y*] sampled is occupied, the removal of the G-actin is evaluated based on the value $\phantom {\dot {i}\!}P^{-}=e^{-E_{1}^{T}}$, with $E_{1}^{T}$ being the sum of the energy of all the links affecting this molecule and (b) at the pointed end of the polar F-actin filament, depolymerization is favored by releasing the bond between the last G-actin and the rest of the polymer, due to destabilization of the link between the last monomer of an F-actin and the rest of the filament. Therefore, the last G-actin in any F-actin is maintained at the cortex only by its link to the inner cell membrane, with energy *E*_1_. Experimental studies [92] estimate the bonds mediated by ATP hydrolysis on the order of 30 kJ/mol (RT = 2.47 kJ/mol), so we can estimate that *E*_0_≈12 in units of RT. This makes the value computed to evaluate removing a G-actin inside a F-actin ($\phantom {\dot {i}\!}P^{-}=e^{-E_{0}-E_{1}}$) around 5 orders of magnitude less probable than removing the last G-actin in a filament ($\phantom {\dot {i}\!}P^{-}=e^{-E_{1}}$). Based on these estimations, the probability of breaking a filament is set as 0, for simplicity.

#### Actin crosslinkers (ACs)

F-actin molecules can cross-link or aggregate in the form of networks or bundles [93]. This aggregation is mediated by actin crosslinkers (ACs) such as fascin, the Arp2/3 complex, Fimbrin, filamin, and *α*-actinin [94–97].

In brief, ACs introduce inter-filament links via a reversible interaction between two actin monomers that are part of adjacent filaments. The binding of ACs stabilizes the F-actin and drives the formation of structures of parallel and anti-parallel F-actin in the form of organized bundles or networks, which are at the core of many biological processes involving actomyosin, such as cell shape and cell movement. The concentration of ACs has been shown to dramatically alter the elastic properties of bundles and networks [98].

The incorporation and release of ACs is implemented as follows: (a) if the position sampled [ *x,y*] is empty and located between two parallel, anti-parallel or perpendicular F-actin polymers, the insertion of an AC molecule is calculated based on the value $\phantom {\dot {i}\!}P^{+}=e^{\mu _{2}}$ and the metropolis method; (b) if incorporation is accepted, a link of energy *E*_2_ is established between the AC and each of the G-actin molecules; (c) when the position sampled [ *x,y*] is already occupied by an AC molecule, its removal is accepted or rejected using $\phantom {\dot {i}\!}P^{-}=e^{-2 \cdot E_{2}}$ and the Metropolis method. Therefore, the strength of *E*_2_ can be modulated to produce stronger or weaker networks of F-actin; and (d) removal of G-actin that are linked to an AC molecule is now $\phantom {\dot {i}\!}P^{-}=e^{-E_{0}-E_{1}-E_{2}}$, so ACs also contribute to stabilization of the filaments, as it occurs in vivo.

#### Myosin

Myosin motors constitute a highly conserved family of molecules that are the major driving force in cell motility, muscle contraction, and transport at the intra-cellular level [68]. Among them, myosin II (also known as conventional myosin) is the responsible for the generation of mechanical force in actin-based cell motility [99]. In nonmuscle cells, active myosin is commonly found as bipolar filaments (anti-parallel arrays of myosin molecules) of 10 to 30 myosin II dimers. These myofilaments interact with F-actin by hydrolyzing ATPs and transforming the chemical energy into mechanical force, allowing their heads to tether F-actin and induce movement, contraction, and tension [100] in the network [101, 102] and in the cell membrane [103, 104].

Myosin and its effect in F-actin networks is introduced in the model as follows: (a) if the position sample [*x,y*] is empty and located between two anti-parallel F-actin polymers, the insertion of a myosin is computed based on the value $\phantom {\dot {i}\!}P^{+}=e^{\mu _{3}}$ (Metropolis method); (b) if incorporation is accepted, a link of energy *E*_3_ is established between the molecule and each of the G-Actin molecules attached to it; (c) when the position sampled [ *x,y*] is already occupied by a myosin molecule, its removal is accepted or rejected using $\phantom {\dot {i}\!}P^{-}=e^{-2 \cdot E_{3}}$ and the Metropolis method; and (d) removal of G-actin linked to a myosin molecule is now $\phantom {\dot {i}\!}P^{-}=e^{-E_{0}-E_{1}-E_{3}}$, so myosin molecules also contribute to stabilization of the filaments.

The effect of myosin depends strongly on the orientation of two F-actin molecules attached to it (summarized in Additional file 12: Fig. S7). In brief, if F-actin molecules are parallel (panel 1), the mechanical action of myosin results simply in the translocation of the molecular motor across the filaments. If the F-actin molecules are anti-parallel (panel 2), the mechanical action of myosin is known to induce sliding [105] of the two F-actin molecules relative to each other.

Since F-actin molecules in the cortex are attached to the inner cell membrane, and therefore cannot slide freely (panel 3), the mechanical force of myosin over anti-parallel F-actin molecules is translated into tension in the filaments and in the network, affecting the shape of the membrane [106]. Tension in the actomyosin has been shown to induce network reorganization [107], network disassembly [73, 108], and F-actin ruptures [107]. This process has been studied extensively and has been shown to have a pivotal role in normal cell division [45], in developmental disorders [109], and in cell motility [102, 108, 110]. Moreover, this sensitivity of F-actin to tension is at the core of a highly dynamic tension sensor in cells [111, 112].

This feateus is introduce in our model as follows: (a) once a myosin is attached to two F-actin molecules, it starts to apply force, resulting in an increase in the elastic potential energy in both filaments, due to its motor properties; (b) This energy *E*_load_ for a given filament can be computed following the simple relation:

$$E_{\text{load}} = \sum_{i=1}^{n} W_{3} \times t_{i} $$

being *n* the number of myosin molecules attached to this particular F-actin, *W*_3_ is the power of a single myosin motor (measured in units of [RT/s]), and *t*_*i*_ is the time since each myosin *i* remains attached to the filament (a single myosin is able to hydrolyze many ATPs and, therefore, apply multiple rounds of mechanical force over the molecules linked to it). Since ATP is assumed in excess in our system, we simply assume that elastic energy accumulates as long as myosin remains attached to the filaments; (c) When this energy *E*_load_ for a given filament is higher than the energy of the link with the inner cell membrane *E*_threshold_ (see F-actin section above), the links between actin and the molecules in the inner cell membrane do not hold and the filament detaches from the cortex [113]; (d) Detached F-actin depolymerizes and free monomers return to the cytoplasm (panel 4 in Additional file 12: Fig. S7); (e) Following detachment of an F-actin (*E*_load_ > *E*_threshold_), the load that was supported by the filament is now supported by the rest of the filaments that remain attached. This is implemented by direct homogeneous redistribution of *E*_load_ to the filaments that remain in the network.

### Graphical representation

To visualize the results, the system is represented as a 2D square grid, below a finite 3D reservoir. In the grid, each molecule type is represented as a short segment with its corresponding orientation and position. Green segments represent G-actin, red for myosin and blue for ACs. On top of the grid, we represent the reservoir as a finite volume, where molecules (now as dots for optimal visualization) are located while not in the cortex.

Although the model does not explicitly accounts for the processes that occur at the cytoplasm (such as diffusion and collisions), direct visualization of the remaining molecules in the finite reservoir molecules allows us to illustrate this particular characteristic of our-semi-grad canonical approach. This is important because the amount of remaining molecules that are not in the grid strongly affect the dynamics of the system (the potential function depends on this value, i.e., the more molecules available in reservoir, the likelihood of insertion increases).

Since these free molecules are assumed to diffuse much faster than the dynamics in the cortex, molecules in the reservoir are simply represented randomly positioned in the 3D volume, for the sake of simplicity. Additional file 11: Fig. S6A is a simplified representation of the system, with the previous features highlighted.

Figures related to F-actin polymerization are plotted based on the effective value of the potential function, defined as:

$$<\mu_{1}> = \mu_{1,0} + \gamma_{1}*\text{log} (N_{1,0}) $$

### Experiments

#### *Drosophila* stocks and genetics

In this study, we use the following *Drosophila* stocks and molecular tools UAS- *α*PS1;UAS- *β*PS [114], the follicle stem cell driver traffic jam-Gal4 (tj-gal4, [115]), and the ubiquitin- lifeactinGFP construct (Ubi-LifeActGFP, [58]). To analyze F-actin distribution and dynamics in UAS- *α*PS1;UAS- *β*PS overexpressed FCs, tj-Gal4;Ubi-LifeActGFP females were crossed to UAS- *α*PS1;UAS- *β*PS males. All stocks and crosses were maintained at 25 ^∘^C.

#### Time-lapse image acquisition

For live imaging, 1–2-day-old females are fattened on yeast at 25 ^∘^C for 48–96 h before dissection. Culture conditions and time-lapse microscopy are performed as described in [116]. Ovarioles are isolated from the ovaries dissected in supplemented Schneider medium (GIBCO-BRL). Movies are acquired on a Leica SP5 MP-AOBS confocal microscope equipped with a 40 × 1, 3 PL APO oil objective, and Leica hybrid detectors (standard mode). Frames are taken every 30 s up to 1 h. For each frame, eleven to twelve *Z*-stacks, with a 0.42 *μ*m interval, are captured to cover the entire basal surface of the cells.

#### Image processing and data analysis

For quantification of basal actin dynamics over time, maximal projections of confocal stacks are created to account for egg chamber curvature. Integrated intensity of actin are quantified from manually selected regions using the ImageJ software. Background value taken from cell-free region was subtracted from all data series. Data are subjected to Gaussian smoothening with *s* = 3. A moving average filter (second order) was applied to remove low-amplitude noise. For quantification of the integrin intensity along actin filaments, maximal projections of confocal stacks are produced to cover the entire basal actin organization. First, the region of interest (ROI) occupied by actin filaments is outlined by hand in each cell. ROIs are then transferred to the integrins fluorescence channel. The mean fluorescent intensity of integrins by pixel is calculated dividing integrated intensity by the total area occupied by actin filaments, using the ImageJ software.

#### Immunohistochemistry

Flies are grown at 25 ^∘^C and yeasted for 2 days at 25 ^∘^C before dissection. The ovaries are dissected from adult females at room temperature in Schneider’s medium to preserve the cytoskeletal structures (Sigma Aldrich). Fixation is performed incubating the egg chambers for 20 min with 4% paraformaldehyde in PBS (ChemCruz). Samples are then permeabilized using phosphate-buffered saline (PBT) + 0.1% Tween 20. The following primary antibodies were used: chicken anti-GFP and mouse anti- *β*PS (1/50, DHSB, Iowa). Fluorescence-conjugated antibodies used were Alexa Fluor 488 and Alexa Fluor 561 (Life Technologies). Samples are mounted in Vectashield (Vector Laboratories) and imaged on a Leica Stellaris equipped with a 40 × 1, 3 HC PL APO CS2 oil objective.

## Supplementary Information

Additional file 1: **Movie S1.** Time-lapse movie of the system with F-actin forming and treadmilling in the grid.

**Additional file 2** **Figure S1.** Percentage of G-Actin in filaments. Percentage of G-Actin in filaments of different size for the three characteristic regimes.

**Additional file 3** **Figure S2.** Dependence of speed of treadmiling with F-actin length. Plot of the dependence of the instantaneous speed on the filament size. Short filaments move faster than average. Long filaments move at the same speed in average.

Additional file 4: **Movie S2.** Time-lapse movie of the system with F-actin and ACs. G-actin is labeled in green, ACs labeled in blue.

**Additional file 5** **Figure S3.** G-Actin, F-actin after incorporation of ACs. Number of G-Actin in the grid (green), G-actin as part of networks (orange) and ACs (blue) after incorporation of ACs into the system at t=5E6 iterations. The formation of networks is initial very fast, followed by a regime where incorporation of molecules into networks is gradually slowing down until equilibrium is reached.

Additional file 6: **Movie S3.** Time-lapse movie of the system showing oscillations. Time-lapse movie of the system showing periodic assembly and disassembly of the actomyosin cortex (G-actin labeled in green, ACs labeled in red, Myosin labeled in red).

**Additional file 7** **Figure S4.** Oscillations for different levels of Myosin. Oscillations in the number of G-actin in the cortex for conditions of (A) low and (B) high concentration of Myosin in the system. The corresponding Fourier transform for each oscillation is also shown.

Additional file 8: **Movie S4.** Oscillations in *D. Melanogaster* basal follicle cells. Control conditions. Cells are stained with Lifeact-GFP.

Additional file 9: **Movie S5.** Oscillations in *D. Melanogaster* basal follicle cells. Experimental conditions. Time-lapse movie of the *D. Melanogaster* basal follicle cells stained with Lifeact-GFP in conditions of over-expression of the two subunits of the integrin molecule.

**Additional file 10** **Figure S5.** Oscillations at lower resolution for different energies. Oscillations of the model at lower resolution, at different energies *E*=1 (left) and *E*=5 (right). When resolution is lowered, the lower part of the oscillation is filtered out and the increase in energy results in a net increase in the average levels of the actin in cortex, more similar to the effect observed experimentally (Fig. 6 F).

**Additional file 11** **Figure S6.** Scheme of the framework. (A) Molecules diffuse freely in a three-dimensional space (cytoplasm) adjacent to a two-dimensional grid (inner plasma membrane) where molecules can attach. G-Actin (green) molecules in the grid interact and polymerize directionally to form F-Actin. ACs (blue) and Myosin (red) also interact with F-Actin to form networks of F-actin. (B) F-actin filament is formed by assembly at the barbed end (regulated by *μ*_1_) and disassembly at the pointed end (regulated by *E*_1_). (C) Linker formation of ACs and Myosin to F-Actin are regulated by *μ*_2_ and *μ*_3_, respectively. Release of ACs and Myosin is regulated by *E*_2_ and *E*_3_, respectively. (D-F) Shape of the potential function *μ*_*i*_ at a given time point for different values of (D) the reference potential *μ*_*i*,0_, (E) the total G-actin molecules in the system *N*_*i*,0_, and (F) the shape parameter *γ*_*i*_.

**Additional file 12** **Figure S7.** Scheme of the effect of Myosin over F-actin. (1), Myosin movement in parallel f-Actin. (2) F-actin sliding. (3) Tension building in the filaments. (4) Release from cortex after threshold tension is reached. After release, tension (illustrated in red) is redistributed to other F-actin in the network.

## Data Availability

Code is available in the following reference to the Zenodo repository [117].
