# Supplementary figures and images for: A coarse-grained approach to model the dynamics of the actomyosin cortex
Source: BMC Biol. 2022 Apr 22;20:90. doi: 10.1186/s12915-022-01279-2 (PMC9034637; doi:10.1186/s12915-022-01279-2)

### Nucleation Phase

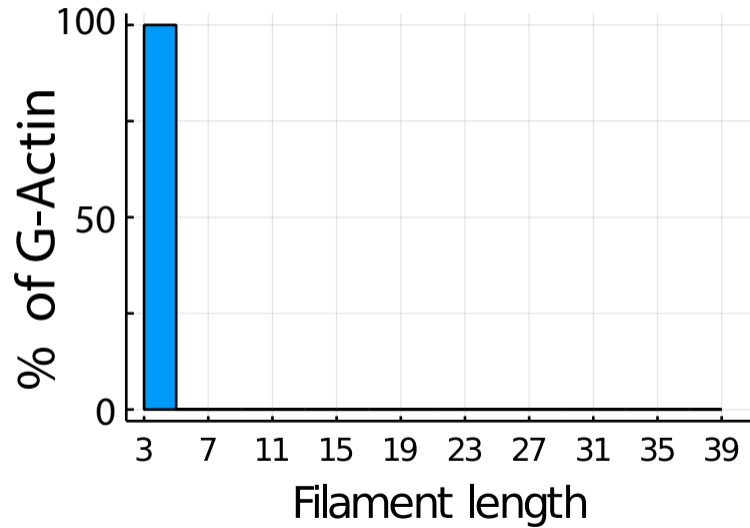

### Linear Phase

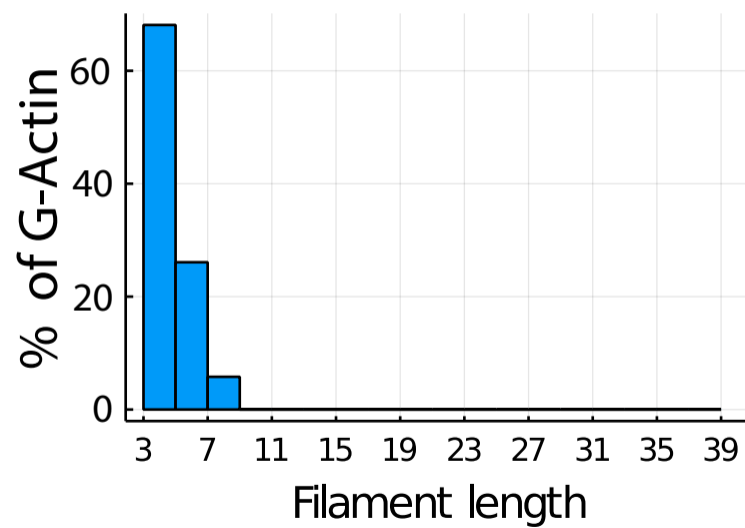

### Equilibrium Phase

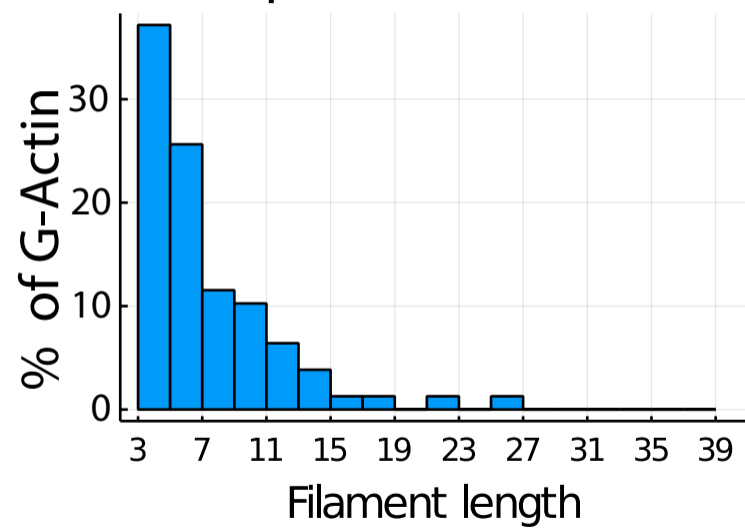

Supplement: Supplementary file 2 — Additional file 2 Figure S1. Percentage of G-Actin in filaments. Percentage of G-Actin in filaments of different size for the three characteristic regimes. [file 12915_2022_1279_MOESM2_ESM.pdf]

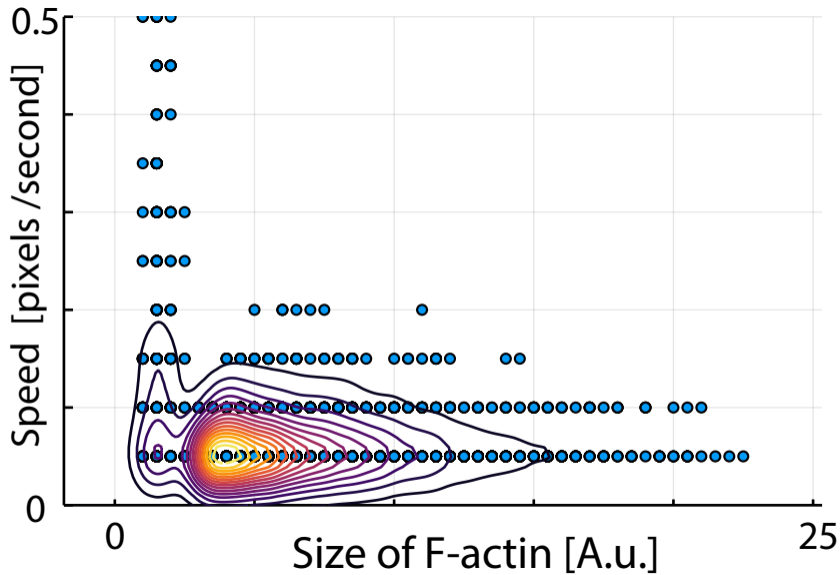

Supplement: Supplementary file 3 — Additional file 3 Figure S2. Dependence of speed of treadmiling with F-actin length. Plot of the dependence of the instantaneous speed on the filament size. Short filaments move faster than average. Long filaments move at the same speed in average. [file 12915_2022_1279_MOESM3_ESM.pdf]

**(A)**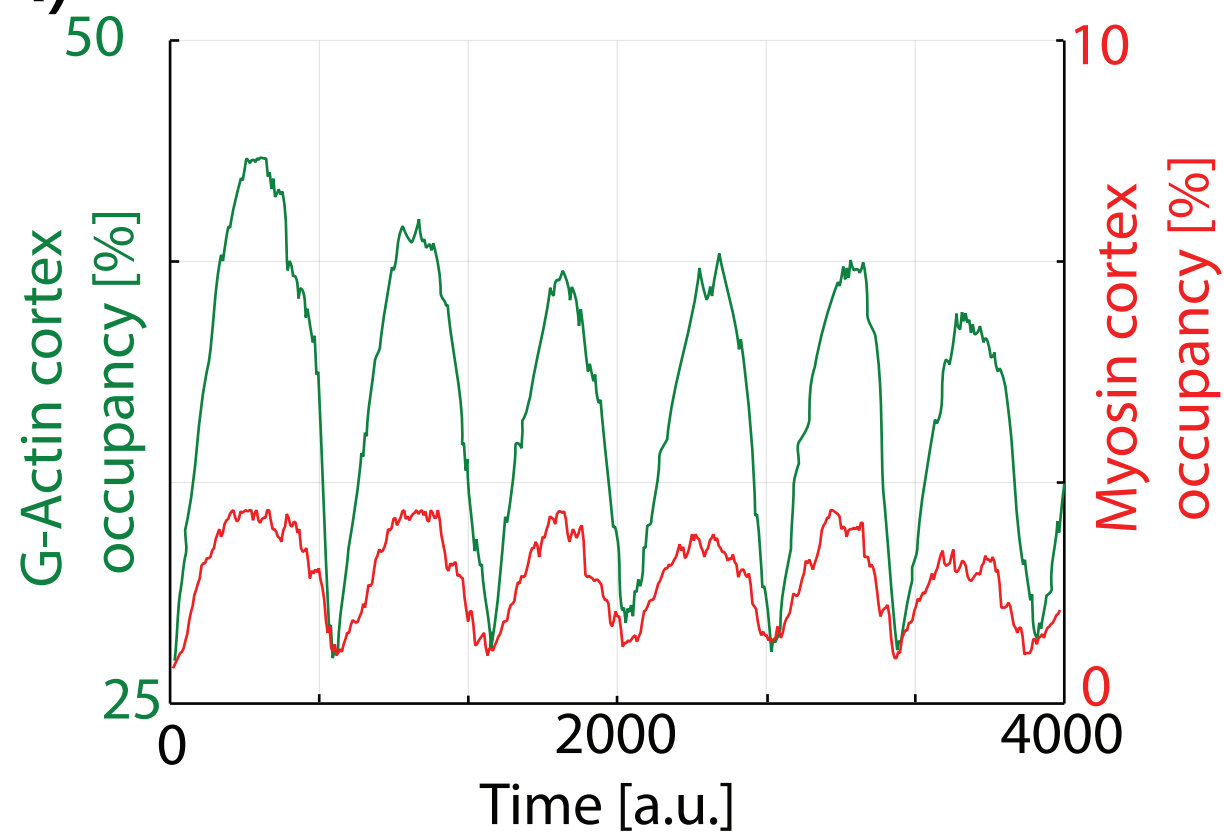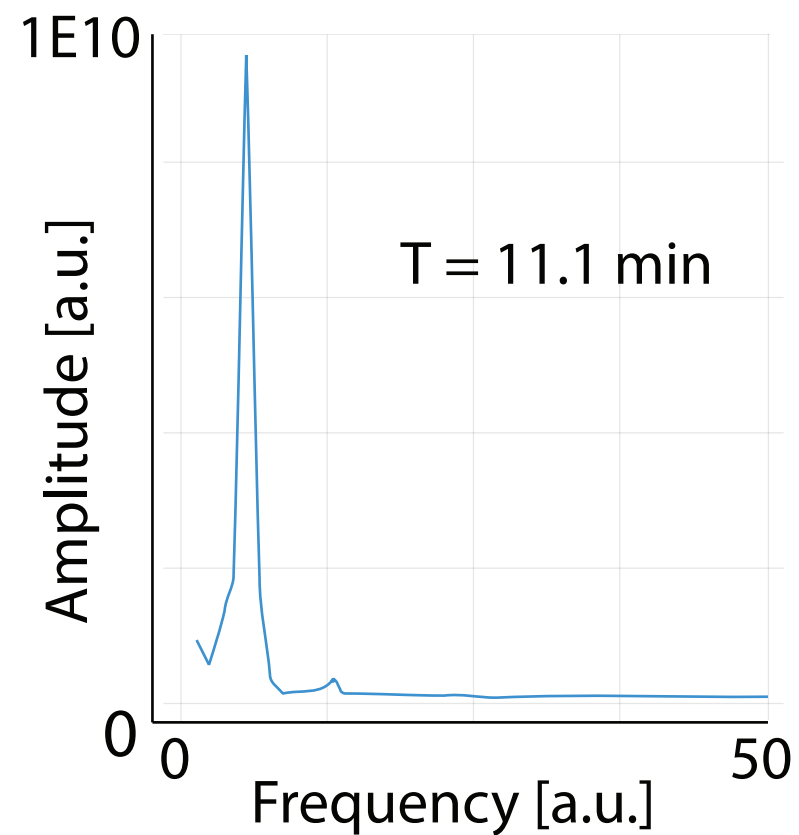**(B)**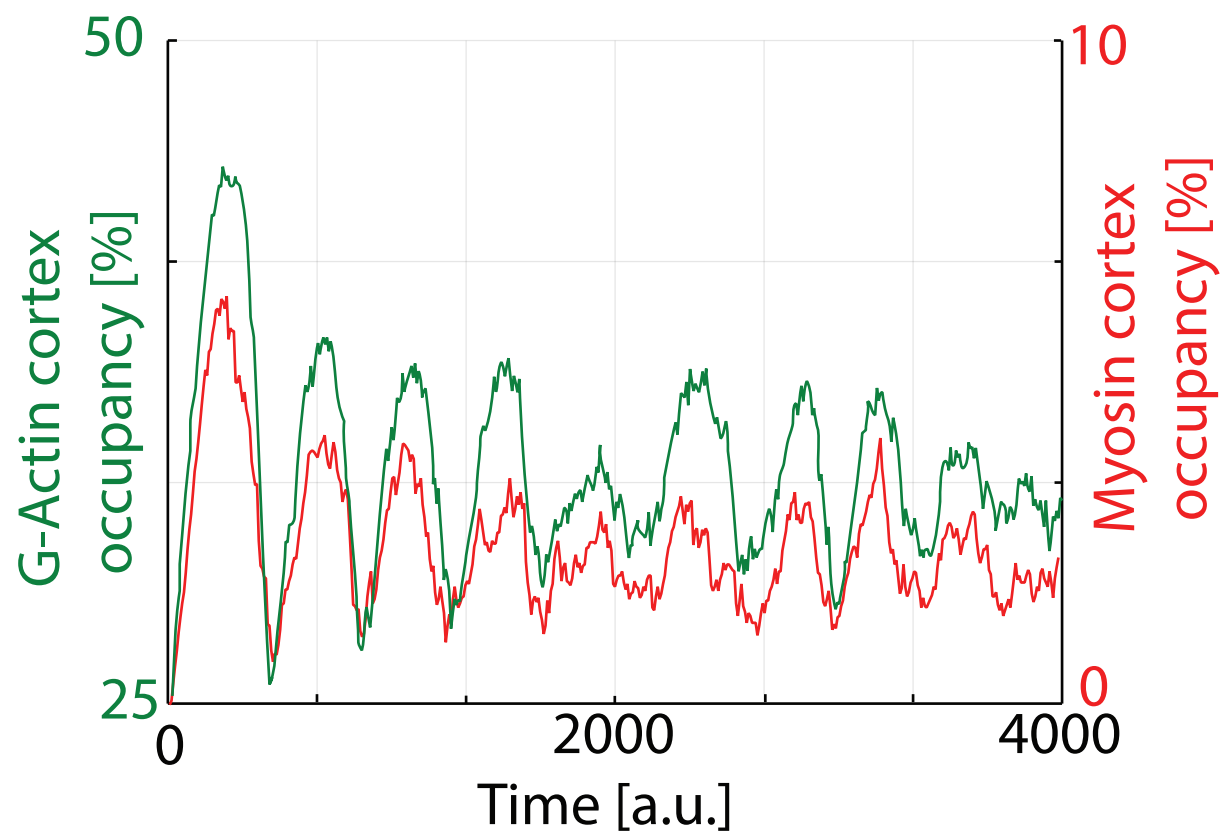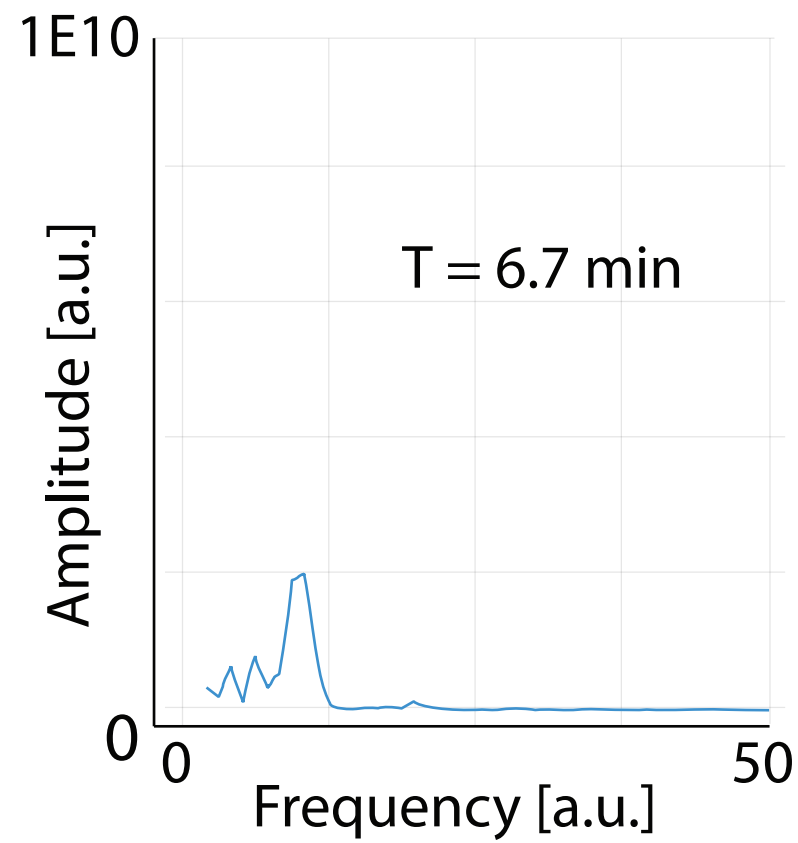

Supplement: Supplementary file 7 — Additional file 7 Figure S4. Oscillations for different levels of Myosin. Oscillations in the number of G-actin in the cortex for conditions of (A) low and (B) high concentration of Myosin in the system. The corresponding Fourier transform for each oscillation is also shown. [file 12915_2022_1279_MOESM7_ESM.pdf]

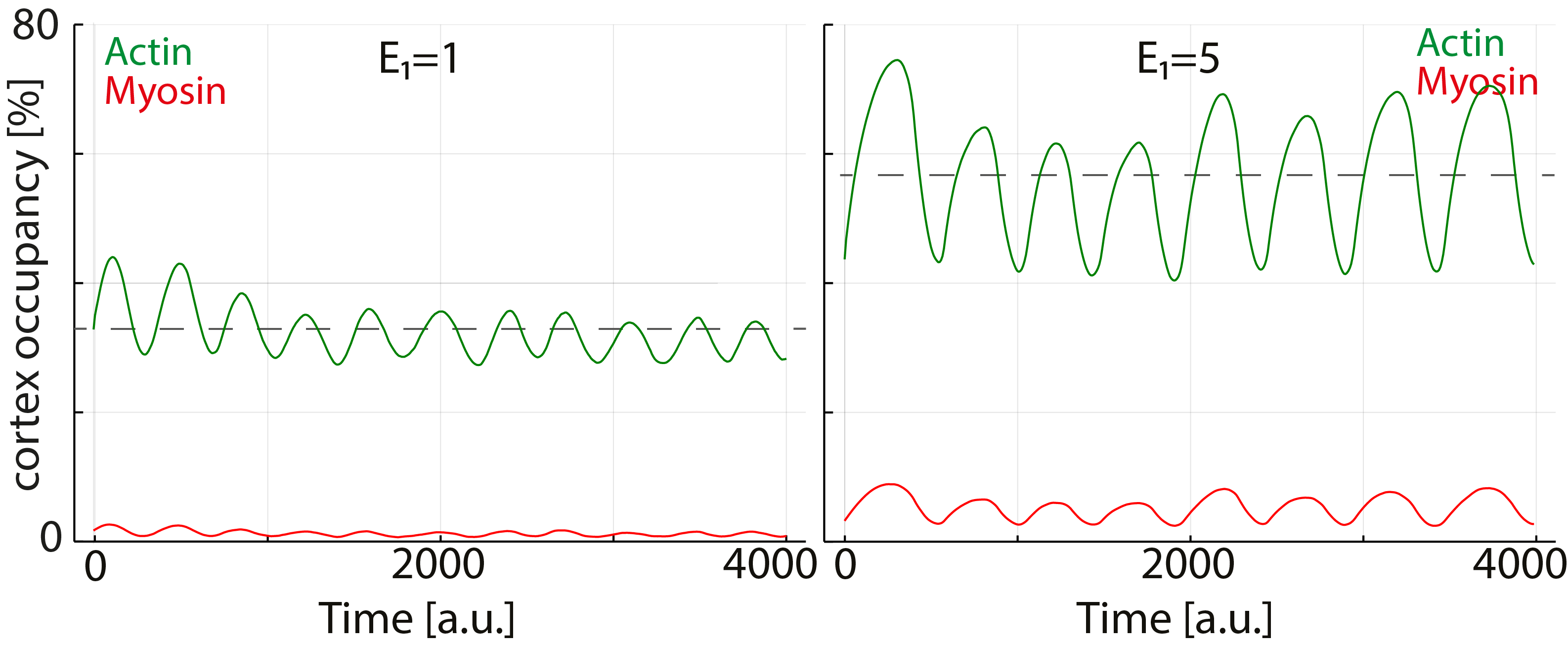

Supplement: Supplementary file 10 — Additional file 10 Figure S5. Oscillations at lower resolution for different energies. Oscillations of the model at lower resolution, at different energies E=1 (left) and E=5 (right). When resolution is lowered, the lower part of the oscillation is filtered out and the increase in energy results in a net increase in the average levels of the actin in cortex, more similar to the effect observed experimentally (Fig. 6 F). [file 12915_2022_1279_MOESM10_ESM.png]

**(A)**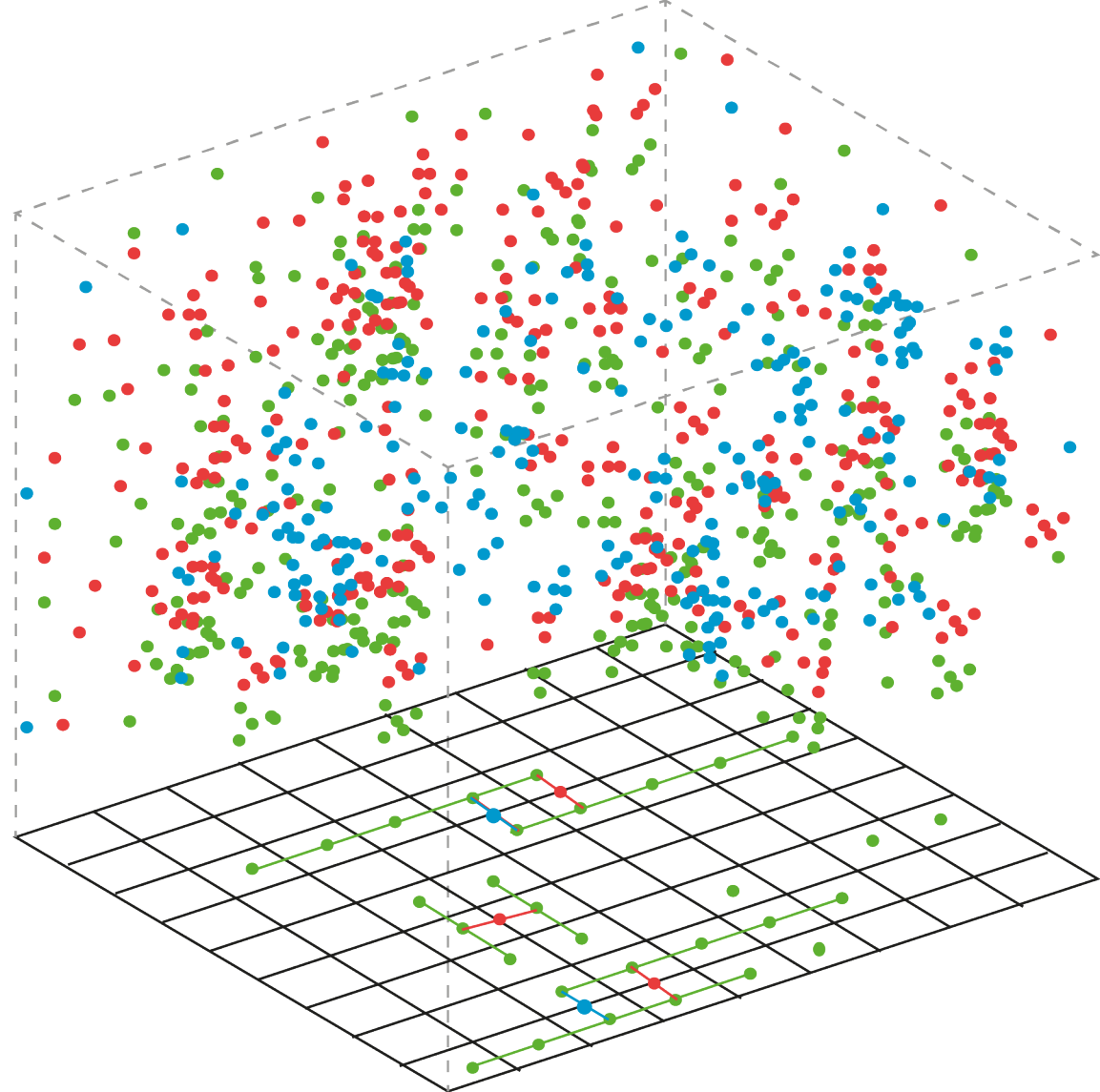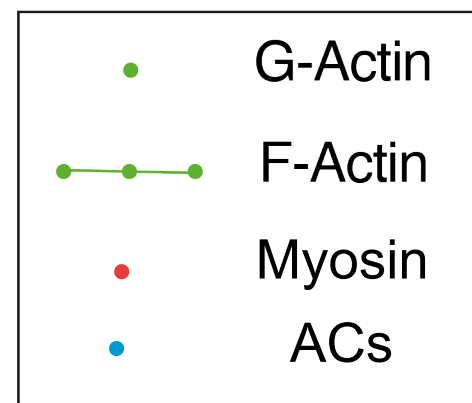**(B)**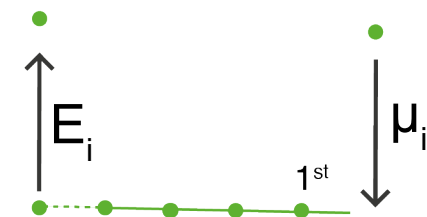**(C)**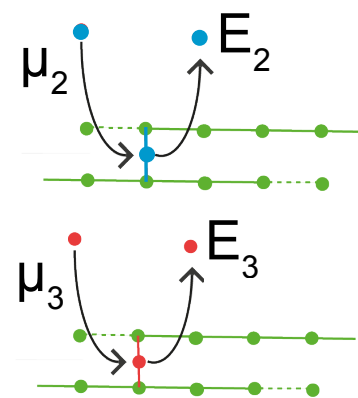**(D)**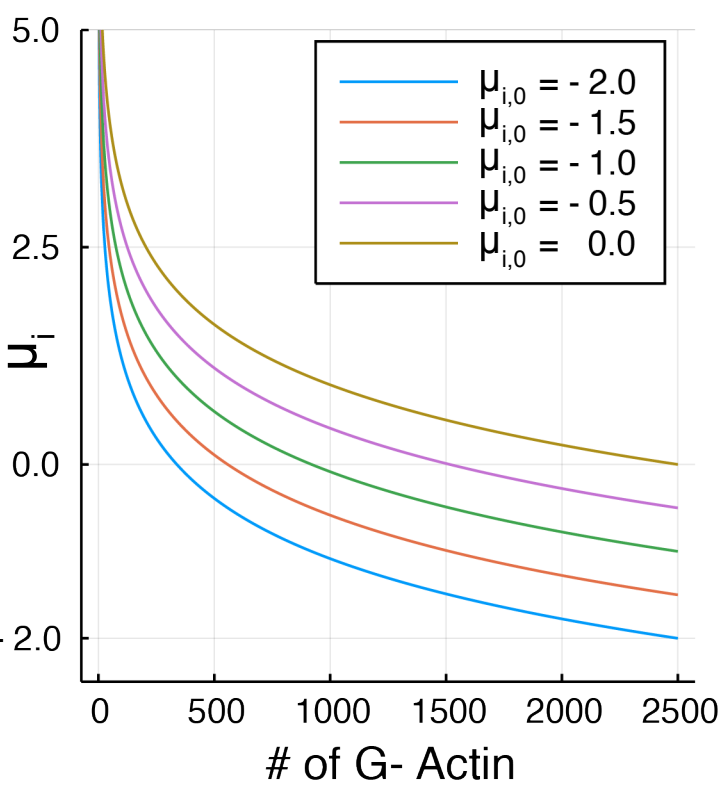**(E)**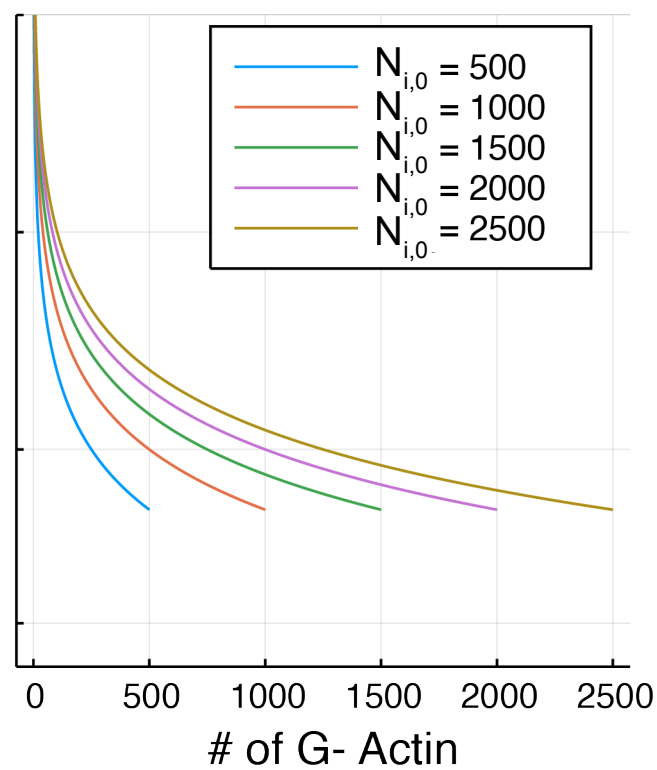**(F)**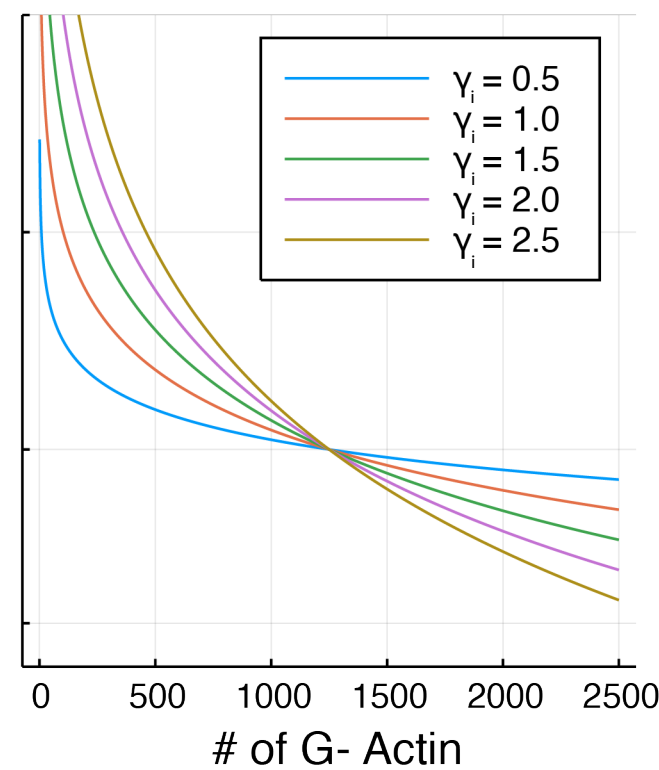

Supplement: Supplementary file 11 — Additional file 11 Figure S6. Scheme of the framework. (A) Molecules diffuse freely in a three-dimensional space (cytoplasm) adjacent to a two-dimensional grid (inner plasma membrane) where molecules can attach. G-Actin (green) molecules in the grid interact and polymerize directionally to form F-Actin. ACs (blue) and Myosin (red) also interact with F-Actin to form networks of F-actin. (B) F-actin filament is formed by assembly at the barbed end (regulated by μ1) and disassembly at the pointed end (regulated by E1). (C) Linker formation of ACs and Myosin to F-Actin are regulated by μ2 and μ3, respectively. Release of ACs and Myosin is regulated by E2 and E3, respectively. (D-F) Shape of the potential function μi at a given time point for different values of (D) the reference potential μi,0, (E) the total G-actin molecules in the system Ni,0, and (F) the shape parameter γi. [file 12915_2022_1279_MOESM11_ESM.pdf]
