# Supplementary material for: A coarse-grained approach to model the dynamics of the actomyosin cortex
Source: BMC Biol. 2022 Apr 22;20:90. doi: 10.1186/s12915-022-01279-2 (PMC9034637; doi:10.1186/s12915-022-01279-2)
Supplement: Supplementary file 5 — Additional file 5 Figure S3. G-Actin, F-actin after incorporation of ACs. Number of G-Actin in the grid (green), G-actin as part of networks (orange) and ACs (blue) after incorporation of ACs into the system at t=5E6 iterations. The formation of networks is initial very fast, followed by a regime where incorporation of molecules into networks is gradually slowing down until equilibrium is reached. [file 12915_2022_1279_MOESM5_ESM.pdf]

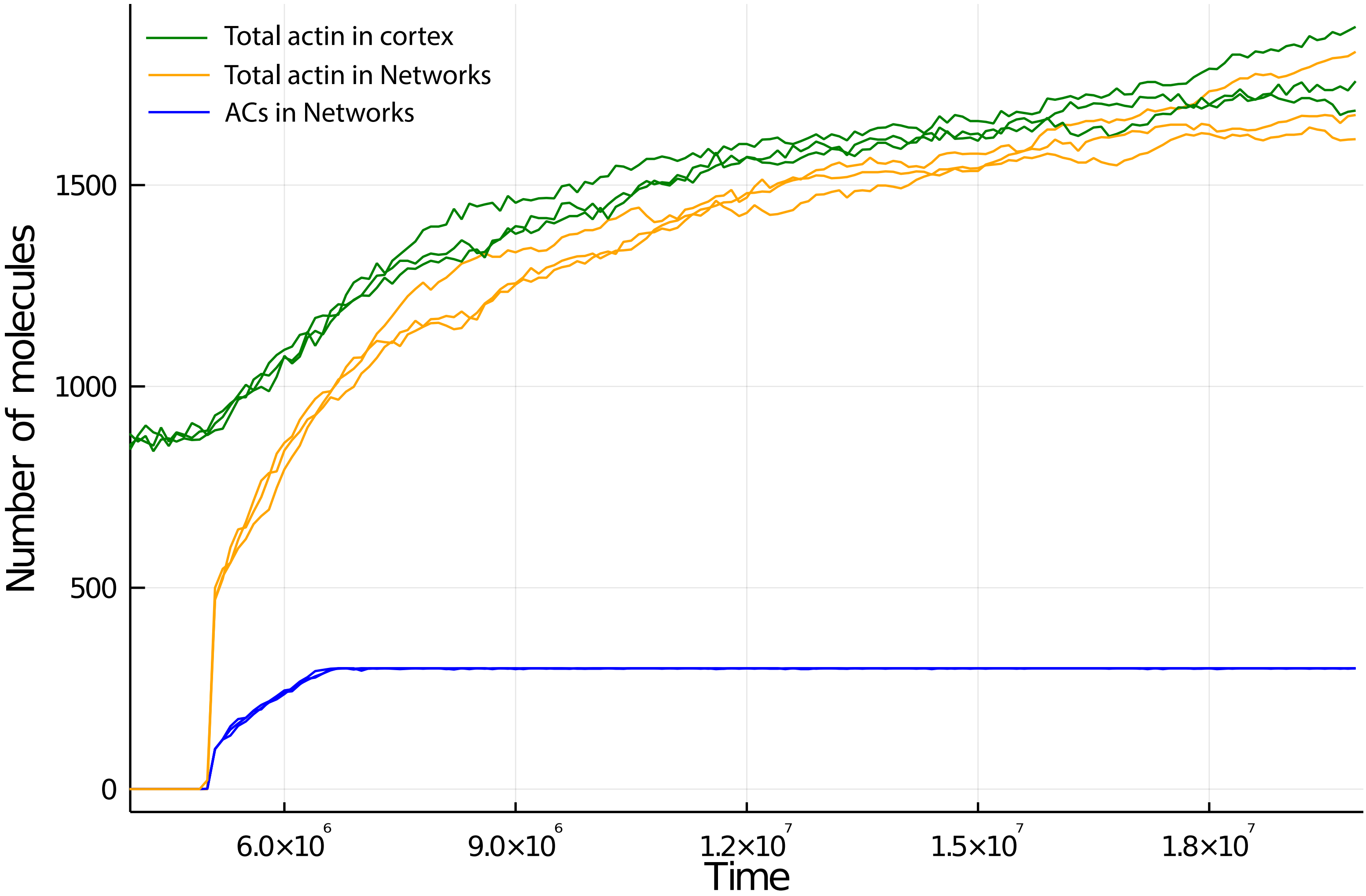
Total actin in cortex
Total actin in Networks
ACs in Networks
Number of molecules
0
500
1000
1500
Time
6.0×10^{6}
9.0×10^{6}
1.2×10^{7}
1.5×10^{7}
1.8×10^{7}
