# Supplementary material for: A coarse-grained approach to model the dynamics of the actomyosin cortex
Source: BMC Biol. 2022 Apr 22;20:90. doi: 10.1186/s12915-022-01279-2 (PMC9034637; doi:10.1186/s12915-022-01279-2)
Supplement: Supplementary file 12 — Additional file 12 Figure S7. Scheme of the effect of Myosin over F-actin. (1), Myosin movement in parallel f-Actin. (2) F-actin sliding. (3) Tension building in the filaments. (4) Release from cortex after threshold tension is reached. After release, tension (illustrated in red) is redistributed to other F-actin in the network. [file 12915_2022_1279_MOESM12_ESM.pdf]

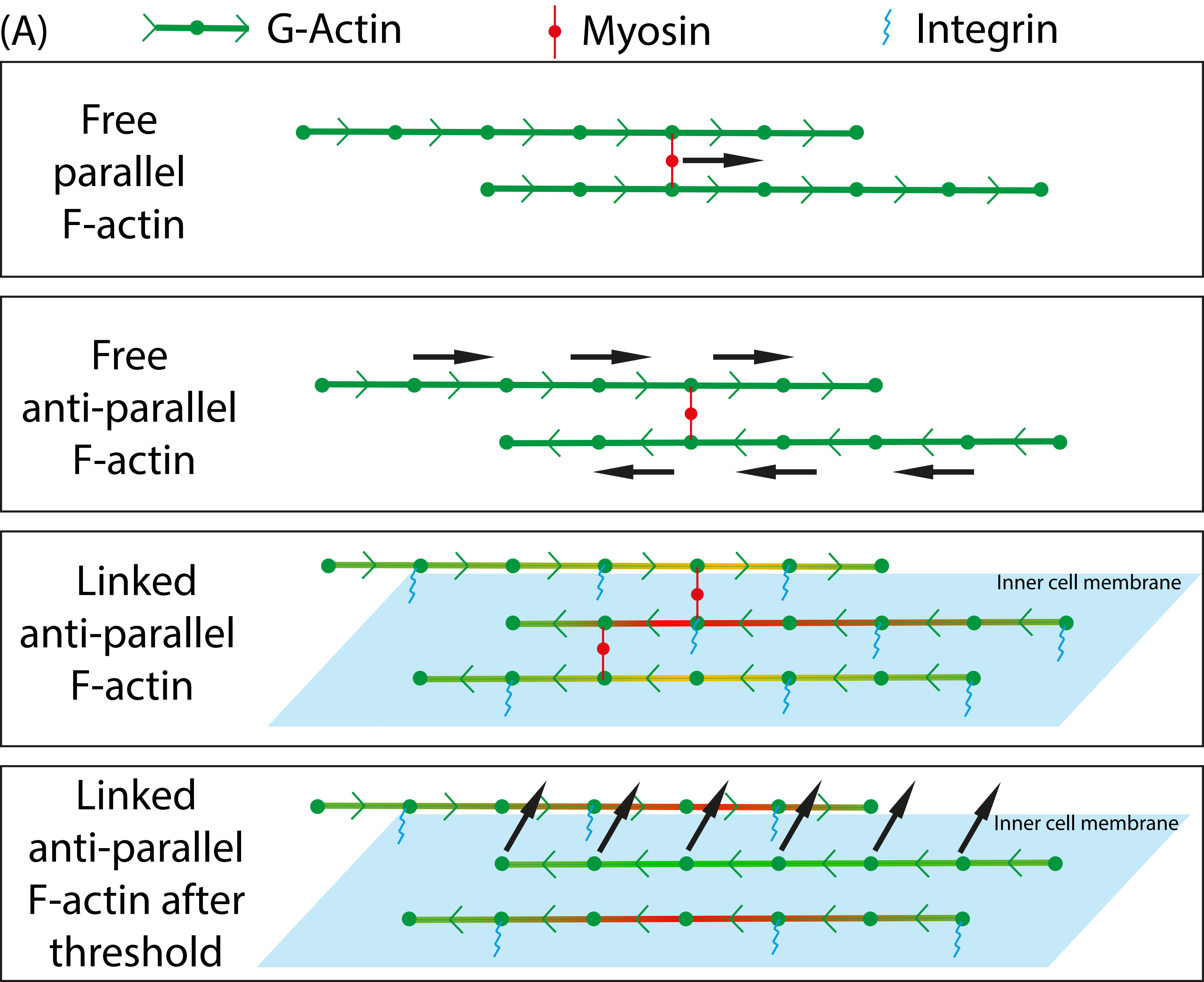
(A)
G-Actin
Myosin
Integrin
Free parallel F-actin
Free anti-parallel F-actin
Linked anti-parallel F-actin
Inner cell membrane
Linked anti-parallel F-actin after threshold
Inner cell membrane
